# Model uncertainties do not affect observed patterns of species richness in the Amazon

**DOI:** 10.1371/journal.pone.0183785

**Published:** 2017-10-12

**Authors:** Lilian Patrícia Sales, Olívia Viana Neves, Paulo De Marco, Rafael Loyola

**Affiliations:** 1 Programa de Pós-graduação em Ecologia e Evolução, Universidade Federal de Goiás, Goiânia, Goiás, Brazil; 2 Departamento de Ecologia, Universidade Federal de Goiás, Goiânia, Goiás, Brazil; 3 Brazilian Research Network on Climate Change–Rede Clima. Instituto Nacional de Pesquisas Espaciais, São José dos Campos, São Paulo, Brazil; University of Colorado, UNITED STATES

## Abstract

**Background:**

Climate change is arguably a major threat to biodiversity conservation and there are several methods to assess its impacts on species potential distribution. Yet the extent to which different approaches on species distribution modeling affect species richness patterns at biogeographical scale is however unaddressed in literature. In this paper, we verified if the expected responses to climate change in biogeographical scale—patterns of species richness and species vulnerability to climate change—are affected by the inputs used to model and project species distribution.

**Methods:**

We modeled the distribution of 288 vertebrate species (amphibians, birds and mammals), all endemic to the Amazon basin, using different combinations of the following inputs known to affect the outcome of species distribution models (SDMs): 1) biological data type, 2) modeling methods, 3) greenhouse gas emission scenarios and 4) climate forecasts. We calculated uncertainty with a hierarchical ANOVA in which those different inputs were considered factors.

**Results:**

The greatest source of variation was the modeling method. Model performance interacted with data type and modeling method. Absolute values of variation on suitable climate area were not equal among predictions, but some biological patterns were still consistent. All models predicted losses on the area that is climatically suitable for species, especially for amphibians and primates. All models also indicated a current East-western gradient on endemic species richness, from the Andes foot downstream the Amazon river. Again, all models predicted future movements of species upwards the Andes mountains and overall species richness losses.

**Conclusions:**

From a methodological perspective, our work highlights that SDMs are a useful tool for assessing impacts of climate change on biodiversity. Uncertainty exists but biological patterns are still evident at large spatial scales. As modeling methods are the greatest source of variation, choosing the appropriate statistics according to the study objective is also essential for estimating the impacts of climate change on species distribution. Yet from a conservation perspective, we show that Amazon endemic fauna is potentially vulnerable to climate change, due to expected reductions on suitable climate area. Climate-driven faunal movements are predicted towards the Andes mountains, which might work as climate refugia for migrating species.

## Background

Climate change is arguably a major threat to biodiversity [1–3]. Loss of suitable climatic conditions may potentially lead to expressive contractions on species and ecosystems distribution as we know so far [2,3]. In synergism with other stressors, such as deforestation, climate change may lead to negative feedbacks in ecosystem resilience [4,5] and increase extinction risk for a large number of species, during and beyond the 21^st^ century [6–8].

Traditionally, the most widely used method to assess the effects of climate change on species distribution are the so-called “species distribution models”–SDMs [9–11]. The SDMs appraise how climatic conditions drive species distribution, at broad spatial scales [12,13]. Although stablished in literature as one of the most useful tools to analyze impacts of climate change on species distribution, SDMs are inherently fraught with uncertainties [9,12,14]. Uncertainties on SDMs emerge as mathematical modeling methods can provide dramatically different responses [12]. Also, the climate forecasts, or coupled Atmosphere-Ocean General Circulation Models–AOGCMs, may lead to distinct results, as well as should distinct greenhouse gas emission scenarios [1,15].

In addition, the two most widely used sources of species distribution data, i.e. occurrence records and maps of extent of occurrence (henceforth range maps; Elith and Leathwick [9]), may lead to potentially different model outcomes. Point-locality records should intuitively have greater reliability than range maps, because the exact location of each species record is known. Data deficiency nevertheless arises for many species, especially those inhabiting remote places [16]. For those species, SDMs fitted with distributional data based on range-maps could supposedly provide an initial understanding of habitat preferences, to be later improved with data refinement [17–19].

Although species-specific responses to climate change may vary with different sources of uncertainty in model predictions, some patterns are emerging globally. Impacts of climate change on biodiversity seem to be trait-mediated and to rely on taxon-related vulnerability [20,21]. Changes not only on population size and reproduction dynamics [22], but also on community composition [23] and species interactions [24] are predicted worldwide. As regards to their geographical distribution, endemic species with small ranges are expected to be the most vulnerable to risks from climate change [25]. Poleward and/or upslope climate-driven faunal migrations are also predicted as temperatures rise [26–28]. If species are unable to keep pace with their suitable climate, or to adapt *in situ*, climate change may lead to global extinction thresholds, at least for some vulnerable taxa [2,29].

Tropical species can be particularly threatened by climate change [30–32]. Species inhabiting equatorial zones experience climate conditions closer to animals upper thermal physiological thresholds [31,33], which are highly preserved among lineages [34]. Even small temperature increases might therefore have negative effects on tropical species’ long-term persistence. As temperature raises, climate-driven faunal movements of tropical species towards more suitable areas, i.e. climate refugia, are also constrained by dispersal limitation and restricted to the upslope direction [35,36]. In this work, we assessed the expected effect of climate change on species’ suitable climate areas, and on overall species richness. We used a potentially threatened biota–the Amazon basin endemic fauna of amphibians, birds and mammals–as our study case. Uncertainties and knowledge gaps are a major issue on assessing the impacts of climate change on biodiversity [37], especially for species-rich regions such as the Amazonia [32]. Enormous extensions of primary tropical forests and road access constraints create geographic bias on occurrence records of Amazonian biodiversity and also on climate data [38,39].

The main goal of this paper was to test whether the expected responses to climate change, in terms of species geographic distribution, are affected by the most recognized sources of uncertainty on SDMs. Specifically, we tested whether: 1) the patterns of species-specific shifts on suitable climate area vary among ensembles of species distribution models calibrated with different types of inputs; 2) the predicted impacts of climate change are uneven across different taxonomic groups. 3) the species richness patterns at biogeographical scale, which emerge from models of species distribution projected in future climate forecasts, are affected by the different combination of inputs in SDMs.

## Methods

### Distribution data

**Fig 1:**
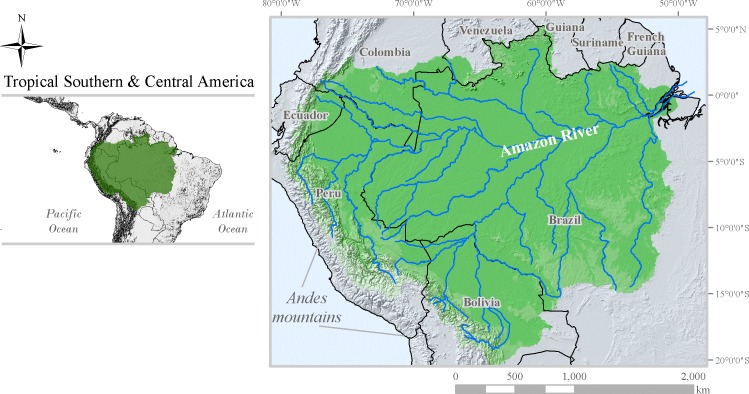
The Amazon basin. Formed by the Amazon River and its tributaries, the Amazon basin spans over nine countries of South America, from the Eastern foot of the Andean mountains towards the Atlantic Ocean. The extent of our study area is also depicted (in the smaller picture) as the Tropical Southern & Central America. Shapefiles of the Amazon River basin were obtained from the Oak Ridge National Laboratory Distributed Active Archive Center [45].

We chose to use the basin of the Amazon river as case study because the origin of Amazonian biodiversity is attributed to river dynamics. Also, Andean post-lift drainage patterns [40,41] contributed to create current Amazonian biodiversity scenario, which is biogeographically intricate with the geologic history of the Amazon basin. Furthermore, stream and river ecosystems (“riverscapes”) are suggested as natural units to which conservation efforts should be targeted [42]. Using the Amazon river basin as a conservation unit target could be potentially useful, as the Amazonia spams over nine countries and, therefore, is not delimited by political borders (Fig 1). The choice of modeling endemic vertebrates was due to a trade-off between the conservation value usually attributed to endemics [43], coupled with threat projections [25], and data availability, which is generally greater for flagship groups like the amphibians, birds and mammals [44].

To compare the results obtained with different sources of data on species occurrence, we calibrated SDMs using two of the most widely used data types: IUCN range maps and point-locality records. At first, we downloaded range maps for all terrestrial mammal species in the world from the International Union for Conservation of Nature and Natural Resources (IUCN) database (www.iucnredlist.org). In the absence of an official list of endemics for the Amazon basin, we selected all amphibian, bird and mammal species whose range maps were completely within the Amazon basin territory plus a 200km surrounding buffer. The identification of endemics based on pre-existing range maps allows to surpass shortcomings on biogeographical data, such as incomplete or biased knowledge of a species distribution based on its occurrence records, and is considered useful for large-scale modeling studies [46,47]. Adding a 200km buffer around the Amazon territory also allows incorporating uncertainties on borders of the IUCN range maps.

The extent of a study area, for species distribution modeling purposes, ideally should only include areas that are accessible for species. Accessible areas, in this case, are those within reach of species dispersal from a biogeographical perspective [48]. To accommodate that restriction, we chose a background representative of all climate conditions that Amazon endemic species probably experienced and/or were restricted to by biogeographical history. Our extent therefore is delimited by a new biogeographical regionalization–the Tropical Southern & Central America (Fig 1)–in which the Neotropical region is splitted into a core tropical and a temperate portion [49]. After defining the study area extent, the species range maps were overlapped into a fishnet-like square grid, in an equal area projection (Albers equal area conic), covering the full extent of the Tropical Southern & Central America (xmn = -85, xmx = -33, ymn = -33, ymx = 22). For each species, all cells with more than 50% of their area overlapped by a range map were attributed as “presences”. Cells outside range maps were considered “absences”, thus creating a presence-absence-like matrix [henceforth PA-matrix]. Only species with more than 30 “presences” in the PA- matrix were considered in this work.

The choice of the grid resolution used here (25 x 25 Km²) aimed at maximizing the reliability of climate information, while assuming moderate commission error. Because there are few weather stations in low populated areas such as the Amazonia, the WorldClim interpolated data for those regions is based on small satellite information [39]. Uncertainty is higher and climate predictions are therefore less accurate for those areas compared to other well sampled regions. Although there are very high resolution climate data available for the Amazon region, these data are likely unreliable for fine resolution analysis [39]. Had we chosen to use a finer resolution (10 x 10 Km², for example) would imply assuming higher levels of uncertainty while falsely giving the impression of a fine resolution. Also, inferences from range maps at very fine resolutions are known to overestimate the area of occupancy of individual species and lead to erroneous patterns of species richness [50]. To allow for quantitative comparisons (please see Methods sub-section “Uncertainty analysis”) between model results from range-maps *vs* point-locality data, we also needed that both data were in the same resolution.

We are aware that using a 25 x 25 Km² grid to convert range maps to a PA-matrix probably inflates commission error (all cells within a species’ range are converted into “presences”, although the actual species distribution may be uneven across its range [50]), even though the use of such resolution is common practice in SDM literature [18,51–54]. However, upscaling point-locality occurrence information to coarser scales (e.g. 100 x 100 Km²) also results in commission error, because regions as far as 100km from where the species was found are likewise included as “presences”. In this study, gridding was necessary for comparison between model outcomes, so that we needed to use the same resolution for models calibrated with both types of distribution data (range maps and point-localities). Because our main goal is comparative, we caution that our results should not be interpreted individually.

After defining the species that are endemic to the Amazon basin, we also downloaded point-locality records for those species to compare results from range-map-based *vs* point-locality-based SDMs. These records were downloaded from three virtual databases: the Global Biodiversity Information Facility (GBIF; www.gbif.org); the SpeciesLink project (www.splink.cria.org.br); and the VertNet database (www.vertnet.org). All these sources are freely available online. In addition to those records, we had access to a dataset from the Instituto Chico Mendes para Conservação da Biodiversidade (*Chico Mendes Institute for Biodiversity Conservation*–ICMBio); the official national parks agency in Brazil. Whenever possible in each website search, we included only records previously checked by experts. All these records were individually assessed.

For records that were located more than 200km distant from the IUCN range map border, a literature survey was conducted to verify if new distribution limits were recently defined for species. When there was no evidence of new valid records, all occurrences more than 200km apart from IUCN range map border were removed from our dataset. By doing so, we assumed a conservative approach and only relatively reliable records were maintained. At the same time, removing records distant from the known occupied areas prevented highly updated–but possibly inaccurate–information on species distribution. Another fishnet-like grid with the same extent and resolution was created for point-locality records. Grid cells containing at least one record were considered as “presence” and cells without records as “absence”. Only species with more than 30 records in the grid were modeled, which resulted in a sample size of 288 species. Spatial autocorrelation was accounted for by removing duplicate records on each cell (one “presence” is attributed to a cell regardless one or more records are found). Although there are other methods to account for spatial and environmental autocorrelation, these methods usually only refer to point-locality and abundance data, not to data from range maps [55,56]. As our main goal is comparative, we therefore chose to maintain the same structure for all models.

### Climate data

Climate data for current conditions were produced by interpolation of weather stations information from years 1960–1990 [39] and are available in the WorldClim database (www.worldclim.org/current). For selecting from all bioclimatic variables available in WorldClim, we created a pairwise correlation matrix. From the least-correlated variables (cutoff = 0.6), we selected those though to be biologically relevant for our study. Because tropical species experience relatively constant temperatures–compared to temperate species–which are usually close to animal’s upper safety thresholds [31], we chose variables related to temperature range and heat extremes. Water availability is associated with thermoregulatory behavior, which can alleviate climate effects on organisms [33], so we also selected variables related to precipitation on hostile periods, such as the driest month and the warmest quarter. Variables used for species distribution modeling in this work therefore were: Mean Diurnal Range (Bio 2); Temperature Seasonality (Bio 4), Mean Temperature of Warmest Quarter (Bio 10), Precipitation of Driest Month (Bio 14), Precipitation of Warmest Quarter (Bio18). Prior to variable selection, the WorldClim dataset was clipped to our study extent and re-scaled for our resolution, by averaging neighboring values (function = mean), using the function *lets*.*presab* in *letsR* package [57]. All variable selection analysis were performed using the *caret* package [58].

We used future climate scenarios developed by the Fifth Assessment Report (AR5) of The Intergovernmental Panel on Climate Change (IPCC), also available from WorldClim (www.worldclim.org/cmip5_30s). We used climate models for year 2070 due to our focus on uncertainty sources, once differences among projections increase from year 2050 on [1]. Future climate projections are based on expectations of greenhouse gas emission rates derived from anthropogenic actions. Predicted values of temperature and precipitation vary with different emission scenarios. In this work, we included two different scenarios (representative concentration pathways *rcp26* and *rcp85*), which represent extreme expectations of greenhouse gas emission rates. The *rcp26* is an “optimistic” stringent mitigation projection, and *rcp85* is a “pessimistic” or baseline scenario without additional efforts to constrain emissions [1]. Within these extreme scenarios, uncertainties also arise because different climate forecasts, or Atmosphere-Ocean General Circulation Models (AOGCMs), can project distinct climatic conditions for certain areas [59]. So we chose five representatives of each climate forecast known to predict divergent changes in temperature and precipitation [60], to include a wide range of different predictions. The chosen AOGCMs were BCC-CSM1.1 (BC), GFDL-CM3 (GF), HadGEM2-ES (HE), CCSM4 (CC), MIROC-ESM (MR).

### Species distribution modeling

Species point-localities or range maps data, coupled with climate predictors, were used to model species potential distribution and to assess the effects of climate change on species future distribution. We fitted those data to nine different modeling methods, and performed ensembles exclusively among methods known to be statistically and conceptually similar, according to Rangel and Loyola’s [59] classification scheme. In that classification, distance-based or “envelope” are considered the simplest modelling methods, which assume that species geographic distribution is constrained by climatic tolerances. Regression-based or “statistical” methods can fit a larger number of parameters to different types of relationships between species occurrence and environmental variables. Machine-learning methods, the most complex algorithms in this classification, attempt to maximize the relationship between occurrences and predictors, while minimizing the number of parameters.

Those three main groups of algorithms were used in this work and their representatives were: 1) Envelope methods (bioclimatic models–BIOCLIM, Euclidian distance, ecological niche factor analysis–ENFA); 2) Statistical methods (generalized linear models–GLM, generalized additive models–GAM, multivariate adaptive regression splines–MARS); 3) Machine-learning methods (random forest–RndFor, artificial neural networks–NNet, and maximum entropy–MaxEnt). By fitting models according to this algorithm grouping approach, we maintain interpretability of ensemble model outputs [61]. For a general description of these methods, please see Franklin [62] and Peterson et al. [10]. The same kind of pseudo-absence data was randomly chosen and used in all models (*sensu* de Oliveira [55]), regardless presence-only or presence-absence methods. (Details on model parameterization can be found as supporting information on S1 Table).

We randomly partitioned the “presences” in PA-matrix data into two subsets of calibration or train (75% of data), and validation or test (remaining 25%), and this process was repeated 10 times. Training outputs therefore yielded Grinnellian niche projections on the geographic space [56,63], whereas the test dataset evaluated model performance. Continuous predictions of habitat suitability were converted into binary projections. For doing so, we found the threshold in the relative operating characteristic (ROC curve), with maximum sensitivity (proportion of correctly predicted presences) and specificity (proportion of correctly predicted absences) values. Then, model accuracy was evaluated with the True Skills Statistics (TSS), the measure of choice for binary predictions [64], also obtained from sensitivity and specificity (TSS = Sensitivity + Specificity—1) [62]. Values of TSS range from -1 to +1, in which values close to +1 indicate good model predictions and values equal or smaller than zero are no better than random predictions [64]. Although presence-only methods do not require pseudo-absences in model calibration, accuracy assessment from TSS-like measures thus depend on “absences”, or pseudo-absences. Therefore, pseudo-absences in presence-only methods, such as BIOCLIM, were used to characterize the study background and enable calculation of TSS metrics.

### Ensembles of forecasts

Predictions of species response to climate change are filled with uncertainties [12]. Ensembles of forecasts are expected to produce stronger predictions and are considered a useful framework to account for uncertainties on model projections [12]. We performed model ensembles by weighting each model per TSS performance, to discriminate models in terms of their accuracy. Models with TSS < 0.5 were removed from final ensembles. Ensembling procedures included only conceptually similar methods (envelope *vs* statistical *vs* machine-learning methods) and only data from the same source (point-localities *vs* range maps). Consensus maps of potential distribution for current and future time-periods were then created from the frequency at which species appears in each grid cell. The overlay of *per* species consensus layers resulted in the final species richness maps.

**Fig 2:**
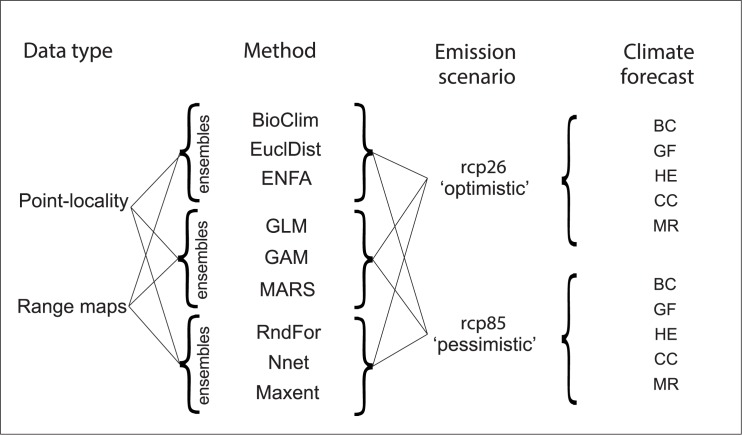
Tree of possibilities. We modeled the distribution of amphibians, birds and mammals that are endemic to the Amazon, using two types of data (point-localities and range maps); three ensembles of envelope (BioClim, EuclidDist, ENFA), statistical (GLM, GAM, MARS) and machine-learning (RndFor, NNet, Maxent) methods. Those models were then projected into five climate forecasts (BC, GF, HE, CC, MR) within two greenhouse gas emission scenarios (*rcp*26 and *rcp*85). Acronyms for methods indicate: BioClim = Bioclimate envelope; EuclidDist = Euclidian distance; ENFA = Ecological niche factor analysis; GLM = generalized linear models; GAM = Generalized additive models; MARS = Multivariate adaptive regression splines; RndFor = Random forest, NNet = Artificial neural networks; Maxent = Maximum entropy. Acronyms for climate forecasts indicate: BC = BCC-CSM1.1; GF = GFDL-CM3; HE = HadGEM2-ES; CC = CCSM4; MR = MIROC-ESM. Representative concentration pathways are represented as *rcp*.

A total of 180 models were fitted for each species (2 datasets X 9 methods X 10 random partitions of PA data). For current conditions, 90 projections of distribution (9 modeling methods X 10 random partitions of PA data) were obtained per species per data type. Ensemble models for current conditions resulted in six projected distributions (2 datasets X 3 ensembles) per species. Further, ensemble models generated 60 future projections for each species (2 datasets X 3 ensembles X 2 climate scenarios X 5 climate models). A visual depiction of those combinations is presented in Fig 2.

### Uncertainty analysis

We aimed at evaluating the impact of different sources of uncertainty on the expected responses of species distribution to climate change. To quantify the relative contribution of such distinct sources of variation among model projections, we performed a hierarchical Analysis of Variance (ANOVA) without replication for each cell [65]. The difference between current projected richness (C) and future projected richness (F) was considered here a proxy of the impact of climate change on species richness (C—F) and was used as response variable.

Data source (range maps and point-locality records), modeling method (BIOCLIM, Euclidian distance, ENFA, RndFor, NNet, MaxEnt, GLM, GAM, MARS), representative concentration pathways (*rcp26* and *rcp85*) and future climate forecasts (AOGCMs: BC, GF, HE, CC, and MR) were considered factors. Then, we obtained the sum of squares attributed to each factor. Variance components were considered the proportion of the sum of squares compared to the total sum of squares, as suggested by Diniz-Filho et al. [12]. By doing so, we could identify regions of the Amazonia associated to high uncertainty and their respective drivers. Consensus maps were produced in Bioensembles software [66]. All figures shown in this paper are original and were created in R and ArcGIS 10.3.1 (ESRI, Redlands, CA, USA).

### Model outcome comparison

To verify if data type and modeling method affected model performance, we compared the TSS values of model ensembles. Finally, we tested whether variation on projected range shifts, i.e. current potential distribution (C) *minus* future potential distribution (F) = projected range shifts (C—F), could be attributed to species’ observed range size. We calculated the standard deviation of the values of range shift estimated by all combinations of the factors cited previously. Our rationale here is that differences among projected range shifts could be a result of the differential sampling of varying range sizes. All differences among model outputs were tested with linear models, using the appropriate link function and family distribution in each case.

## Results

### Range shifts

A total of 288 species were modeled (40 amphibian, 225 bird, and 23 mammal species), 18169 point-locality records were used in the modeling, with an average of 63 records per species, though most species had 30–46 records. Seven taxonomic families of amphibians, 35 of birds, and 9 of mammals were represented in this study (S2 Table). One critically endangered (CR), seven endangered (EN), 11 vulnerable (VU), 19 near threatened (NT) and 250 least concern (LC) species, according to IUCN latest classification [67] were modeled in this study (S2 Table).

**Fig 3:**
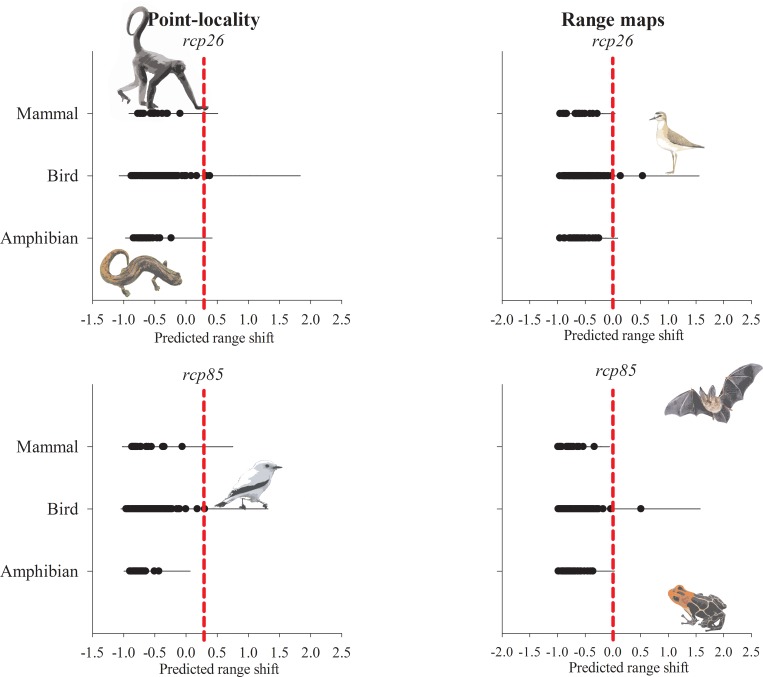
Cumulative variation on predicted climatically suitable areas by taxonomic Family. Horizontal thin bars indicate variation within taxa and black dots are species-specific means on the predicted variation of geographic range (the difference between current and future climatically suitable areas).

Though range shift predictions varied between biological data type, overall model predictions were usually consistent within taxonomic groups (Fig 3). All orders are expected to experience range contractions (S3 Table). At the taxonomic level of family, the same pattern is observed. All vertebrate families are expected to experience range shrinks in both *rcp* scenarios. Range contractions are predicted for most species by all combinations of *rcp* scenarios and biological data types. Overall, amphibians are the group expected to lose the larger climate area (mean = -0.70 ± 0.10), followed by mammals (mean = -0.68 ± 0.16) and birds (mean = -0.59 ± 0.14). Primates were the mammal group most likely to be threatened by climate change. Within birds and amphibians, there was not a dominant group in terms of climate change vulnerability.

In the “optimistic” scenario, five species (*Gastrotheca griswoldi*, *Grallaria przewalskii*, *Rhinella poeppigii*, *Scytalopus acutirostris*, *Stefania evansi*) are predicted to lose all of their climatically suitable areas by at least one ensemble model. In the “pessimistic” scenario, that number raises to 12 species, which are likely to have no climatically suitable area in year 2070 (*Alouatta discolor*, *Amazona kawalli*, *Certhiaxis mustelinus*, *Gastrotheca griswoldi*, *Grallaria przewalskii*, *Odontophorus stellatus*, *Poecilotriccus albifacies*, *Rhinella poeppigii*, *Scytalopus acutirostris*, *Stefania evansi*, *Veniliornis sanguineus*, *Xiphorhynchus spixii)*, according to predictions of at least one ensemble model. Very few species are predicted to experience some range expansion, i.e. two species in the optimistic greenhouse gas scenario and only one in the pessimistic scenario (S3 Table).

### Uncertainty, model fit and species richness patterns

Hierarchical ANOVA showed that the greater source of variation in the effects of climate change on species distribution was the modeling method (median explained variation = 0.50, CI = 0.32–0.66), followed by biological data type (median explained variation = 0.28, CI = 0.07–0.5), climate forecasts (median explained variation = 0.08, CI = 0.05–0.13) and *rcp* scenario (median explained variation = 0.04, CI = 0.002–0.009). Uncertainty patterns varied geographically across the Amazon basin and among the main ANOVA factors (S1 Fig). Total uncertainty seems to be concentrated at Roraima state, in Brazil, and at the Southeastern portion of the Amazon basin, close to the region called the “Arch of Deforestation”. Also, a large amount of variation was depicted to the Andes mountains, especially for climate-related factors (greenhouse gases emission scenarios–*rcp26* and *rcp85 –*and climate forecasts–AOGCMs). Uncertainty attributed to biological data type was mainly aggregated at the center of the Amazon basin. Methods uncertainty was less spatially structured than other factors, although a smooth trend towards peripheral zones of the Amazon basin could be observed.

### Model outcome comparison

**Fig 4:**
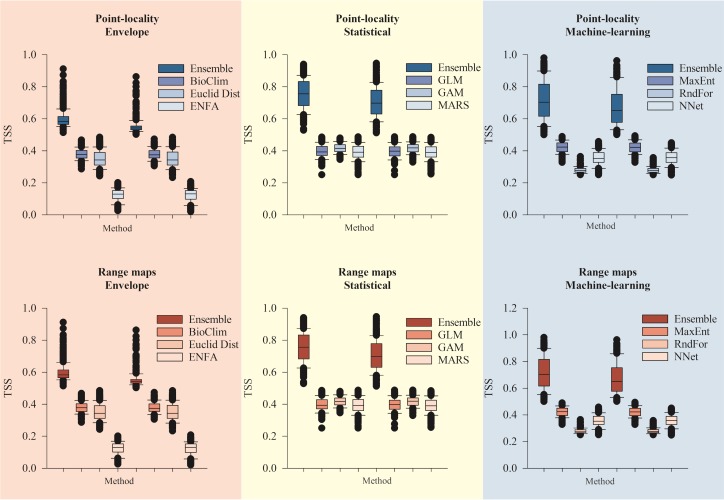
Model fit in relation to biological data and modeling method. Thick bars represent median and 95% confidence interval, dots are outlier values. Ensembles of envelope (bioclimatic models–BIOCLIM, Euclidian distance, ecological niche factor analysis–ENFA); statistical (generalized linear models–GLM, generalized additive models–GAM, multivariate adaptive regression splines–MARS); and machine-learning methods (random forest–RF, artificial neural networks–ANN, and maximum entropy–MaxEnt) were built and their model fit compared to individual methods. True Skills Statistics (TSS) values did not differ neither between biological data source (Point-locality vs Range-map-based models), or ensembles of methods, although they differed among individual modeling methods. Acronyms for methods indicate: BioClim = Bioclimate envelope; EuclidDist = Euclidian distance; ENFA = Ecological niche factor analysis; GLM = Generalized linear models; GAM = Generalized additive models; MARS = Multivariate adaptive regression splines; RndFor = Random forest, NNet = Artificial neural networks; Maxent = Maximum entropy. Acronyms for climate forecasts indicate: BC = BCC-CSM1.1; GF = GFDL-CM3; HE = HadGEM2-ES; CC = CCSM4; MR = MIROC-ESM. Representative concentration pathways are depicted as *rcp*.

Ensembles of methods had consistently better model fit than their respective isolated methods (Fig 4). Model performance (in terms of TSS) exhibited a relationship between data type and modeling method. Both data types had higher TSS values on ensembles of statistical methods (Point-locality: TSS _mean_ = 0.75 ± 0.06, Range-maps: TSS _mean_ = 0.75 ± 0.06) and lower TSS values on ensembles of envelope methods (Point-locality: TSS _mean_ = 0.72 ± 0.05, Range-maps: TSS _mean_ = 0.75 ± 0.06). Machine-learning ensembles, however, produced better results on models calibrated with point-locality records (Point-locality: TSS _mean_ = 0.60 ± 0.06, Range-maps: TSS _mean_ = 0.66 ± 0.06).

Point-locality-based models predicted slightly smaller contractions on climate area, but variation was larger (*rcp26*_mean_ = -0.53 ± 0.22; *rcp85*_mean_ = -0.64 ± 0.19) than range-map-based model outputs (*rcp26*_mean_ = -0.59 ± 0.17; *rcp85*_mean_ = -0.71 ± 0.15). We also found a negative relationship between the species’ observed range size and difference among model outcomes (*F* = 13.886, p = 0.0002, DF = 286). In other words, distribution models for species with smaller range size resulted in more divergent outputs than did those for widely distributed species.

**Fig 5:**
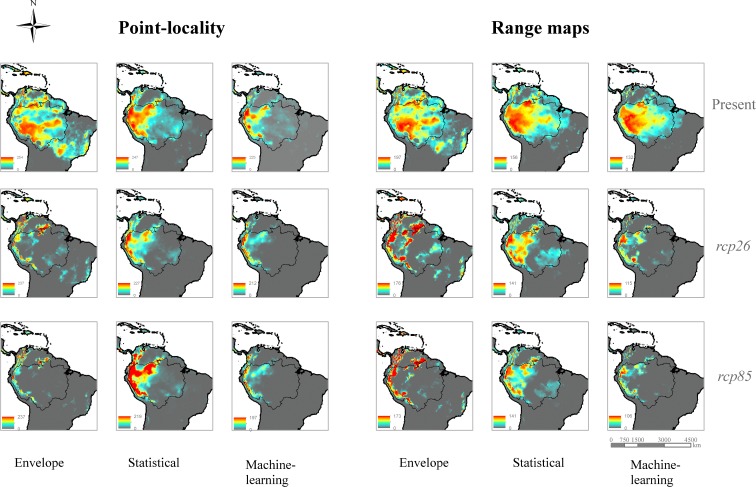
Species richness patterns expected for Amazonia species on year 2070. Based on ensembles of modeling methods (Envelope: BIOCLIM, ENFA, Euclidian distance; Statistical: GLM, GAM, MARS; and Machine-learning: MaxEnt, RF, ANN) projected on two extreme greenhouse gas emission climate scenarios (*rcp*26 and *rcp*85), using two sources of biological data (IUCN range maps and point-locality records).

Although patterns of species richness varied per data source and ensembles of modeling methods, average species richness was expected to be reduced across the entire Amazon basin. For all models, current richness of endemic species was concentrated on the eastern portion of the Amazon basin, in a gradient downstream the Amazon River. All consensus maps predicted that, on year 2070, climatic suitable areas for most species will be more concentrated in the eastern portion of the Amazon basin, moving upwards the Andes mountains, in a comparison with current location of climatic suitable areas for most species (Fig 5). As for present-day species richness patterns, envelope methods predicted higher climate suitability for a larger number of species outside the Amazon area. Those climate areas predicted by envelope methods also include some portions of the Brazilian Atlantic forest. Statistical methods were more “conservative” and predicted smaller range contractions across the Amazon territory in year 2070.

## Discussion

Much has been said about model uncertainty regarding the use of species distribution models (SDMs) to assess climate change effects on the geography of biodiversity [12,15,68,69]. Addressing the impacts of yet-to-come threats to biodiversity is a task inherently fraught with uncertainty. No validation can be performed on future climate models and different data sets and/or modeling methods are expected to produce divergent results [13,16]. Yet the impact of model uncertainties on observed ecological patterns is still obscure. In this paper, we modeled the potential distribution of Amazon endemic species and projected those distributions into future climate, while explicitly incorporating the major factors known to affect the outcome of SDMs. Although predictions were not identical among the many sources of uncertainty addressed in this work, especially when comparing individual modeling methods and data sources, some biological patterns were evident in most model outcomes.

We found the same visual patterns of geographic distribution on species richness of Amazon endemic vertebrates (discussed below) using both kinds of data. Point-locality-based maps revealed the same broad biogeographical patterns as those calibrated with range-map based data. Maps calibrated with both types of data were also similar in terms of expected range shifts. Converting range maps into presence-absence matrices with grid resolutions higher than 1–2 degrees of lat/long supposedly alters biogeographical patterns of species richness [50], although changes in grain size do not seem to affect predictions from SDMs [70]. In this work, we used point-localities and range maps as source of species distribution information for calibrating SDMs at 25 x 25 Km² (0.25 degrees of lat/long) and we found the same biogeographical patterns using both data types. Model outcomes of both data types exhibited similar patterns of species richness and range shifts expected as response to climate change.

Here we do not advocate the indiscriminate use of IUCN range maps as distributional data for all species, but we recognize that range maps could provide an initial knowledge about species niche requirements, especially those that are range restricted, data deficient and/or those that occupy remote places [12]. Our small sample size (n = 288 species) and particularities regarding endemics’ restricted distribution prevent us from making wider generalizations. We do however acknowledge that data deficiency may lead to underestimated conservation efforts and that some biogeographical patterns may be consistent and detectable enough to justify the use of IUCN range maps as distributional data in some cases. However, we also found a negative relationship between the variation on predicted range size and the size of the species’ known distribution. Therefore, predictions are less convergent for geographically restricted species, so our results should be interpreted with caution in those cases.

Methods used to model species distribution can provide such variable outcomes, that simply assessing whether a species’ range may reduce or expand can prove unsuccessful in some cases [71]. In this work, the greatest source of variation on predictions was the modeling method, which has been shown to severely affect the outcome of SDMs [12]. Ensembling among models however improved our predictions and reduced output variation, which was already expected because ensembles of forecast are known to reduce uncertainty related to modeling methods [12]. Differences among outputs of ensemble models were much smaller than those from individual models. Surprisingly, ensembles of statistical methods had better performance than machine-learning and envelope methods. Machine-learning methods are more complex and usually related to more explanatory power [61], in which predictions are best fit to the original data [9]. Machine-learning methods had better performance only for point-locality data, but not for models calibrated with range maps. Once machine-learning methods usually benefits precision but penalizes generality and ability to extrapolate [10,61], we suggest that machine-learning predictions for point-locality data should give more precise but less extrapolatable projections. Envelope methods had the worst performance, probably because they sacrifice predictive power for the sake of generality, contrary to machine-learning methods [61].

The first biogeographical pattern consistent in all consensus maps of species richness was an East-western gradient from the Andes foot downstream the Amazon River. That pattern was already expected because, as mentioned before, the Amazon biodiversity is biogeographically linked to the Amazon River geologic history. The uplift of Andean mountains in the pre-Quaternary reconfigured the previously deltaic Amazon basin drainage patterns, by creating an influx of nutrients and sediments into the basin, and an edaphic mosaic extremely rich in species [41]. The Amazon basin biodiversity therefore results from complex interactions between paleoclimatic and vegetational shifts, in addition to spatial variability on precipitation, especially in East-west but also North-south direction [72]. Our findings highlight that the Western portion of the Andes mountains contains an expressive richness of endemic species, which smoothly diminishes downstream the Amazon River towards the Atlantic Ocean. That East-western pattern on current species richness was consistent across all types of data source and ensembles of methods.

A second pattern we found in this work was species migration upwards the Andes lift. All model projections for year 2070 predict an increase of species richness for the Andean region of the Amazon, followed by reductions across the Amazon basin. As climate changes, species have historically moved towards more suitable climates [35]. Climate-driven faunal movements are successful when directed to environmental habitats that enhance biota survival at spatio-evolutionary timescales [35,73]. Those environments offer the best chances of survival on climate change and are therefore called “climate refugia” [26,73]. Poleward and/or upslope faunal movements as response to recent climate are a trend across the globe [28]. As temperature raises, poleward movements are restricted for tropical species but upslope migrations are expected and have been observed for some groups [27]. Some regions, such as the Amazon basin, are predicted to concentrate high density of faunal movements towards higher altitudes [35]. Our results indicate that Andean slopes may work as climate refugia for Amazon endemic species as upwards climate-driven movements may allow species to track their suitable climate, which has strong applications for Amazon conservation. That uplift movement pattern was also consistent and detectable for all data source types, modeling methods, greenhouse gas emission scenarios and climate forecasts.

Predictions of species’ expected contractions on suitable climate area were also consistent and similar in most model outcomes. Most species are expected to lose some climatic area in the future, a consistent pattern across all data source types, modeling methods, climate scenarios and climate models. Losses are congruent among taxonomic levels and all orders and families are likely to lose expressive amounts of suitable climate for most species. If such species are not able to keep pace with climate change and track their suitable areas or adapt *in situ*, they will probably be highly threatened in the wild [29]. Our results suggest that some groups may lose larger areas of suitable climate and could be more exposed to non-analogue climates.

Amphibians in special were highlighted in this work as more threatened than birds and mammals. The Amazon basin has also been considered a hotspot of endemism of amphibians that are highly vulnerable to climate change [20]. Ectotherms are dependent on the surrounding temperature and most amphibians have a highly permeable skin, in both terrestrial and aquatic life stages. Those eco-morphological features explain amphibians’ sensitivity to environmental change and are probably related to their decline worldwide [74]. Among mammals, our work suggests that climate change impacts will be more deleterious to primates than to any other mammal group, in terms of average predicted losses in suitable climate area. Neotropical primates are essentially poor dispersers for their arboreal habits and canopy-dependence [75], which will probably prevent them to keep pace with climate change [36]. Species that occupy lowland human-modified landscapes may require long migrations in order to access suitable climates and severe reductions in range size have already been predicted for other primates [36].

Although we found some variation on species distribution model outputs, our work suggests that some biogeographical patterns may be detectable and consistent on model outcomes from the traditionally used methods in SDM. Congruence among predictions of different model outcomes and alarming consequences for biodiversity as response to climate change have already been observed for a myriad of taxa and biodiversity levels [2]. Coherence on expected outcomes of models including the major sources of known uncertainties reinforces the usefulness of SDM approaches on assessing biodiversity responses to climate change, meanwhile highlights the need for substantial conservation actions.

## Supporting information

S1 Fig Uncertainty on species richness pattern for Amazonia mammals. Species distribution models projected for year 2070, derived from the hierarchical ANOVA factors: biological data source (IUCN range maps and point-locality records), modeling method (BIOCLIM, ENFA, Euclidian distance, GLM, GAM, MARS, MaxEnt, RF, and ANN), greenhouse gases emission scenarios (rcp26 and rcp85) and future climate simulation models (AOGCs: BC, GF, HE, CC, and MR). Acronyms for methods indicate: BioClim = Bioclimate envelope; EuclidDist = Euclidian distance; ENFA = Ecological niche factor analysis; GLM = generalized linear models; GAM = Generalized additive models; MARS = Multivariate adaptive regression splines; RndFor = Random forest, NNet = Artificial neural networks; Maxent = Maximum entropy. Acronyms for climate forecasts indicate: BC = BCC-CSM1.1; GF = GFDL-CM3; HE = HadGEM2-ES; CC = CCSM4; MR = MIROC-ESM. Representative concentration pathways are represented as rcp.
(TIF)

S1 Table Details on model parameterization. Model parametrization of the nine methods used to model species distribution and evaluate the impacts of climate change on Amazon biodiversity. All methods included the automatic search using ROC as probability cutoff, permutation with 10 cross-validation replicates and 75% replicate training.
(DOCX)

S2 Table Taxonomic information and conservation status of the species used in this work. Acronyms are indicated, as follows: LC: least concern, NT: near threatened, VU: vulnerable, EN: endangered; Decr: decreasing, Sta: stable, Unkn: unknown.
(DOCX)

S3 Table Summary statistics of Amazon endemic amphibians, birds and mammals. Taxonomic information of species for which we estimated vulnerability to climate change is included. The mean range shift is the variation in the number of suitable climate cells in species potential distribution (current potential distribution—future potential distribution) and was calculated for two scenarios of climate change (*rcp*26 and *rcp*85), using IUCN range-maps and point-locality records as input data.
(DOCX)
