# Supplementary figures and images for: Model uncertainties do not affect observed patterns of species richness in the Amazon
Source: PLoS One. 2017 Oct 12;12(10):e0183785. doi: 10.1371/journal.pone.0183785 (PMC5638225; doi:10.1371/journal.pone.0183785)

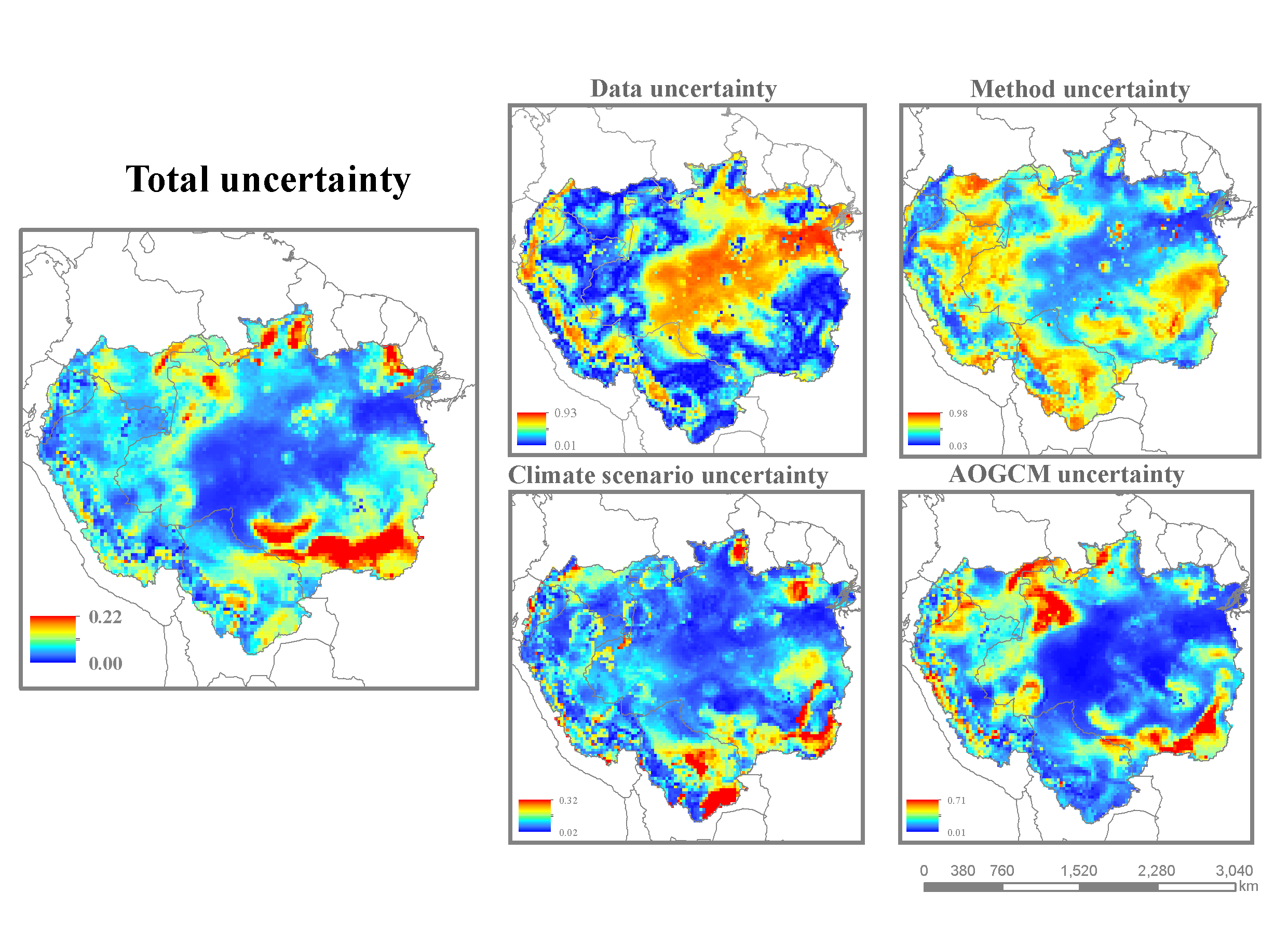

Supplement: S1 Fig — Species distribution models projected for year 2070, derived from the hierarchical ANOVA factors: biological data source (IUCN range maps and point-locality records), modeling method (BIOCLIM, ENFA, Euclidian distance, GLM, GAM, MARS, MaxEnt, RF, and ANN), greenhouse gases emission scenarios (rcp26 and rcp85) and future climate simulation models (AOGCs: BC, GF, HE, CC, and MR). Acronyms for methods indicate: BioClim = Bioclimate envelope; EuclidDist = Euclidian distance; ENFA = Ecological niche factor analysis; GLM = generalized linear models; GAM = Generalized additive models; MARS = Multivariate adaptive regression splines; RndFor = Random forest, NNet = Artificial neural networks; Maxent = Maximum entropy. Acronyms for climate forecasts indicate: BC = BCC-CSM1.1; GF = GFDL-CM3; HE = HadGEM2-ES; CC = CCSM4; MR = MIROC-ESM. Representative concentration pathways are represented as rcp. (TIF) [file pone.0183785.s001.tif]
