# Supplementary material for: Model uncertainties do not affect observed patterns of species richness in the Amazon
Source: PLoS One. 2017 Oct 12;12(10):e0183785. doi: 10.1371/journal.pone.0183785 (PMC5638225; doi:10.1371/journal.pone.0183785)
Supplement: S1 Table — Model parametrization of the nine methods used to model species distribution and evaluate the impacts of climate change on Amazon biodiversity. All methods included the automatic search using ROC as probability cutoff, permutation with 10 cross-validation replicates and 75% replicate training. (DOCX) [file pone.0183785.s002.docx]

**SUPPORTING INFORMATION**

**Supplementary tables**

**S1 Table. Details on model parameterization.** Model parametrization of the nine methods used to model species distribution and evaluate the impacts of climate change on Amazon biodiversity. All methods included the automatic search using ROC as probability cutoff, permutation with 10 cross-validation replicates and 75% replicate training.

| Modeling method | | Data requirement | Description | Specific parameterization |
| --- | --- | --- | --- | --- |
| Envelope | |  |  |  |
|  | BIOCLIM | Presence-only | Predictor values in a grid cell are compared to the distribution percentiles of species’ occurrences to calculate the relative suitability of that grid cell. | None. |
|  | Euclidean distance | Presence-only | The relative suitability of a given cell is the Euclidean distance from the centroid of the environmental envelope to the predictor value for the cell. | None. |
|  | ENFA | Presence-only | Species presence data is compared to the background environment. The mean, standard deviation and covariances of X random points from the background data are calculated for each environmental layer. | None. |
|  |  |  |  |  |
| Statistical |  |  |  |  |
|  | GLM | Presence-absence | Regression-based method that is adaptable to the response variable and accommodates different distributions. | Logit link, binomial distribution. |
|  | GAM | Presence-absence | Similar to GLM but identifies smooth functions that improve model fit. | Three spline degrees |
|  | MARS | Presence-absence | Non-parametric regression similar to GAM but defines variable relationships in local scale. | Basis functions are: constant; articulate (hinge); and the product of two or more functions. |
|  |  |  |  |  |
| Machine-learning | |  |  |  |
|  | Random forest | Presence-absence | Iteratively searches for the best regression tree. Each new tree is a random subset of predictors and their roots. The final output is the average of all trees. | To split in each node, 50 trees and three variables were used. We chose the minimum node size to split as one. |
|  | Artificial neural networks | Presence-absence | Pattern-detection technique, in which classification on each net node is a function describing the non-linear relationship among predictors. | Ten cross validations were used for neural network optimization. |
|  | MAXENT | Presence-background | Based on the concept of relative entropy, i.e. the uncertainty associated to the species occurrence. | Linear, quadratic, and product features were not subjected to regularization. We also did not apply regularization to hinge and threshold features. |
