# Supplementary material for: Model uncertainties do not affect observed patterns of species richness in the Amazon
Source: PLoS One. 2017 Oct 12;12(10):e0183785. doi: 10.1371/journal.pone.0183785 (PMC5638225; doi:10.1371/journal.pone.0183785)
Supplement: S2 Table — Acronyms are indicated, as follows: LC: least concern, NT: near threatened, VU: vulnerable, EN: endangered; Decr: decreasing, Sta: stable, Unkn: unknown. (DOCX) [file pone.0183785.s003.docx]

**SUPPORTING INFORMATION**

**Supplementary tables**

**S2 Table. Taxonomic information and conservation status of the species used in this work.** Acronyms are indicated, as follows: LC: least concern, NT: near threatened, VU: vulnerable, EN: endangered; Decr: decreasing, Sta: stable, Unkn: unknown

| Species name | Family | Status | Population | Description  year | Native countries |
| --- | --- | --- | --- | --- | --- |
| *Aglaeactis castelnaudii* | Trochilidae | LC | Decr | 1848 | Peru |
| *Akodon aerosus* | Cricetidae | LC | Sta | 1913 | Bolivia, Ecuador, Peru |
| *Alouatta discolor* | Atelidae | VU | Decr | 1823 | Brazil |
| *Alouatta puruensis* | Atelidae | LC | Unkn | 1941 | Brazil, Peru |
| *Amazona kawalli* | Psittacidae | NT | Sta | 1989 | Brazil |
| *Ameerega parvula* | Dendrobatidae | LC | Sta | 1882 | Ecuador, Peru |
| *Anabazenops dorsalis* | Furnariidae | LC | Decr | 1880 | Bolivia, Brazil, Colombia, Ecuador, Peru |
| *Anairetes agraphia* | Tyrannidae | LC | Sta | 1919 | Peru |
| *Anairetes alpinus* | Tyrannidae | EN | Decr | 1933 | Bolivia, Peru |
| *Anairetes nigrocristatus* | Tyrannidae | LC | Sta | 1884 | Ecuador, Peru |
| *Ancistrops strigilatus* | Furnariidae | LC | Decr | 1825 | Bolivia, Brazil, Colombia, Ecuador, Peru |
| *Anurolimnas castaneiceps* | Rallidae | LC | Decr | 1868 | Bolivia, Brazil, Colombia, Ecuador, Peru |
| *Anurolimnas fasciatus* | Rallidae | LC | Unkn | 1868 | Brazil, Colombia, Ecuador, Peru |
| *Aotus nigriceps* | Aotidae | LC | Unkn | 1909 | Bolivia, Brazil, Peru |
| *Aotus vociferans* | Aotidae | LC | Decr | 1823 | Brazil, Colombia, Ecuador, Peru |
| *Ara rubrogenys* | Psittacidae | EN | Decr | 1847 | Bolivia |
| *Aratinga weddellii* | Psittacidae | LC | Increasing | 1851 | Bolivia, Brazil, Colombia, Ecuador, Peru |
| *Artibeus anderseni* | Phyllostomidae | LC | Unkn | 1916 | Bolivia, Brazil, Colombia, Ecuador, Peru |
| *Asthenes griseomurina* | Furnariidae | LC | Decr | 1882 | Ecuador, Peru |
| *Asthenes harterti* | Furnariidae | LC | Decr | 1901 | Bolivia |
| *Asthenes helleri* | Furnariidae | VU | Decr | 1923 | Bolivia, Peru |
| *Asthenes humilis* | Furnariidae | LC | Sta | 1873 | Bolivia, Peru |
| *Asthenes maculicauda* | Furnariidae | LC | Decr | 1901 | Argentina, Bolivia, Peru |
| *Asthenes ottonis* | Furnariidae | LC | Sta | 1901 | Peru |
| *Asthenes urubambensis* | Furnariidae | NT | Decr | 1919 | Bolivia, Peru |
| *Asthenes virgata* | Furnariidae | LC | Unkn | 1874 | Peru |
| *Ateles chamek* | Atelidae | EN | Decr | 1812 | Bolivia, Brazil, Peru |
| *Atelopus spumarius* | Bufonidae | VU | Decr | 1871 | Brazil, Colombia, Ecuador, French Guiana, Guyana, Peru, Suriname |
| *Atlapetes canigenis* | Emberizidae | LC | Decr | 1919 | Peru |
| *Atlapetes melanolaemus* | Emberizidae | LC | Decr | 1879 | Peru |
| *Atlapetes rufigenis* | Emberizidae | NT | Decr | 1895 | Peru |
| *Atlapetes rufinucha* | Emberizidae | LC | Decr | 1837 | Bolivia, Peru |
| *Attila citriniventris* | Tyrannidae | LC | Sta | 1859 | Brazil, Colombia, Ecuador, Peru, Venezuela |
| *Aulacorhynchus coeruleicinctis* | Ramphastidae | LC | Decr | 1840 | Bolivia, Peru |
| *Automolus melanopezus* | Furnariidae | LC | Sta | 1858 | Bolivia, Brazil, Colombia, Ecuador, Peru |
| *Boissonneaua matthewsii* | Trochilidae | LC | Unkn | 1847 | Colombia, Ecuador, Peru |
| *Brotogeris sanctithomae* | Psittacidae | LC | Sta | 1776 | Bolivia, Brazil, Colombia, Peru, Ecuador |
| *Callicebus lucifer* | Pitheciidae | LC | Unkn | 1914 | Brazil, Colombia, Ecuador, Peru |
| *Capito aurovirens* | Capitonidae | LC | Decr | 1829 | Brazil, Colombia, Ecuador, Peru |
| *Cebuella pygmaea* | Callitrichidae | LC | Decr | 1823 | Bolivia, Brazil, Colombia, Ecuador, Peru |
| *Cercomacra nigrescens* | Thamnophilidae | LC | Unkn | 1859 | Bolivia, Brazil, Colombia, Ecuador, Frech Guiana, Peru, Suriname |
| *Cercomacra serva* | Thamnophilidae | LC | Sta | 1858 | Bolivia, Brazil, Colombia, Ecuador, Peru |
| *Certhiaxis mustelinus* | Furnariidae | LC | Sta | 1874 | Brazil, Colombia, Peru |
| *Chaetura egregia* | Apodidae | LC | Decr | 1916 | Bolivia, Brazil, Ecuador, Peru |
| *Chalcostigma ruficeps* | Trochilidae | LC | Sta | 1846 | Bolivia, Colombia, Ecuador, Peru |
| *Chalcostigma stanleyi* | Trochilidae | LC | Decr | 1851 | Bolivia, Ecuador, Peru |
| *Chamaeza nobilis* | Formicariidae | LC | Sta | 1855 | Bolivia, Brazil, Colombia, Ecuador, Peru |
| *Chiasmocleis bassleri* | Microhylidae | LC | Sta | 1949 | Bolivia, Brazil, Colombia, Ecuador, Peru |
| *Chiropotes albinasus* | Pitheciidae | EN | Decr | 1848 | Brazil |
| *Chiropotes satanas* | Pitheciidae | CR | Decr | 1807 | Brazil |
| *Chlorospingus parvirostris* | Thraupidae | LC | Decr | 1901 | Bolivia, Colombia, Ecuador, Peru |
| *Cinnycerthia fulva* | Troglodytidae | LC | Decr | 1874 | Bolivia, Peru |
| *Cinnycerthia peruana* | Troglodytidae | LC | Decr | 1873 | Peru |
| *Cnemarchus erythropygius* | Tyrannidae | LC | Sta | 1853 | Bolivia, Colombia, Ecuador, Peru |
| *Coeligena violifer* | Trochilidae | LC | Decr | 1846 | Bolivia |
| *Conioptilon mcilhennyi* | Cotingidae | LC | Sta | 1966 | Bolivia, Brazil, Peru |
| *Conirostrum ferrugineiventre* | Thraupidae | LC | Sta | 1856 | Bolivia, Peru |
| *Conopophaga aurita* | Conopophagidae | LC | Unkn | 1873 | Brazil, Colombia, Ecuador, French Guiana, Guiana, Peru e Suriname |
| *Conopophaga peruviana* | Conopophagidae | LC | Sta | 1856 | Bolivia, Brazil, Ecuador, Peru |
| *Cotinga maynana* | Cotingidae | LC | Decr | 1766 | Bolivia, Brazil, Colombia, Ecuador, Peru |
| *Cranioleuca albicapilla* | Furnariidae | LC | Sta | 1873 | Peru |
| *Cranioleuca antisiensis* | Furnariidae | LC | Sta | 1859 | Ecuador, Peru |
| *Cranioleuca baroni* | Furnariidae | LC | Sta | 1895 | Peru |
| *Cranioleuca marcapatae* | Furnariidae | VU | Decr | 1935 | Peru |
| *Cranioleuca vulpecula* | Furnariidae | LC | Sta | 1866 | Bolivia, Brazil, Ecuador, Peru |
| *Creurgops dentatus* | Thraupidae | LC | Decr | 1876 | Bolivia, Peru |
| *Crypturellus atrocapillus* | Tinamidae | NT | Unkn | 1844 | Bolivia, Brazil, Peru |
| *Crypturellus bartletti* | Tinamidae | LC | Decr | 1873 | Bolivia, Brazil, Ecuador, Peru |
| *Cyanolyca turcosa* | Corvidae | LC | Sta | 1853 | Colombia, Ecuador, Peru |
| *Cyanolyca viridicyanus* | Corvidae | NT | Decr | 1838 | Bolivia, Peru |
| *Cymbilaimus sanctaemariae* | Thamnophilidae | LC | Decr | 1941 | Bolivia, Brazil, Peru |
| *Dendroplex kienerii* | Dendrocolaptidae | NT | Decr | 1934 | Brazil, Colombia, Peru |
| *Dendropsophus bifurcus* | Hylidae | LC | Sta | 1945 | Bolivia, Brazil, Colombia, Ecuador, Peru |
| *Dendropsophus leali* | Hylidae | LC | Sta | 1964 | Bolivia, Brazil, Peru |
| *Dendropsophus leucophyllatus* | Hylidae | LC | Sta | 1783 | Bolivia, Brazil, Colombia, Ecuador, French Guiana, Guyana, Peru, Suriname |
| *Dendropsophus marmoratus* | Hylidae | LC | Sta | 1768 | Bolivia, Brazil, Colombia, Ecuador, French Guiana, Guyana, Peru, Suriname, Venezuela |
| *Dendropsophus rhodopeplus* | Hylidae | LC | Sta | 1858 | Bolivia, Brazil, Colombia, Ecuador, Peru |
| *Dendropsophus sarayacuensis* | Hylidae | LC | Sta | 1935 | Bolivia, Brazil, Colombia, Ecuador, Peru, Venezuela |
| *Dichrozona cincta* | Thamnophilidae | LC | Decr | 1868 | Bolivia, Brazil, Colombia, Ecuador, Peru, Venezuela |
| *Diglossa glauca* | Thraupidae | LC | Decr | 1876 | Bolivia, Colombia, Ecuador, Peru |
| *Diglossa mystacalis* | Thraupidae | LC | Sta | 1846 | Bolivia, Peru |
| *Drymophila devillei* | Thamnophilidae | LC | Sta | 1906 | Bolivia, Brazil, Colombia, Ecuador, Peru |
| *Edalorhina perezi* | Leptodactylidae | LC | Sta | 1870 | Brazil, Colombia, Ecuador, Peru |
| *Elaenia gigas* | Tyrannidae | LC | Increasing | 1871 | Bolivia, Colombia, Ecuador, Peru |
| *Engystomops petersi* | Leptodactylidae | LC | Sta | 1872 | Colombia, Ecuador, Peru |
| *Entomodestes leucotis* | Turdidae | LC | Decr | 1844 | Bolivia, Peru |
| *Epinecrophylla erythrura* | Thamnophilidae | LC | Sta | 1890 | Brazil, Colombia, Ecuador, Peru |
| *Epinecrophylla leucophthalma* | Thamnophilidae | LC | Sta | 1868 | Bolivia, Brazil, Peru |
| *Epinecrophylla ornata* | Thamnophilidae | LC | Sta | 1853 | Bolivia, Brazil, Colombia, Ecuador, Peru |
| *Eriocnemis luciani* | Trochilidae | LC | Sta | 1847 | Colombia, Ecuador |
| *Eubucco versicolor* | Capitonidae | LC | Unkn | 1776 | Bolivia, Peru |
| *Euphonia finschi* | Thraupidae | LC | Sta | 1877 | Brazil, French Guiana, Guyana, Suriname, Venezuela |
| *Euphonia mesochrysa* | Thraupidae | LC | Decr | 1873 | Bolivia, Colombia, Ecuador, Peru |
| *Eutoxeres condamini* | Trochilidae | LC | Decr | 1851 | Bolivia, Colombia, Ecuador, Peru |
| *Furnarius minor* | Furnariidae | LC | Decr | 1858 | Brazil, Colombia, Ecuador, Peru |
| *Galbalcyrhynchus leucotis* | Galbulidae | LC | Sta | 1845 | Brazil, Colombia, Ecuador, Peru |
| *Galbalcyrhynchus purusianus* | Galbulidae | LC | Decr | 1904 | Bolivia, Brazil, Peru |
| *Galbula chalcothorax* | Galbulidae | LC | Decr | 1855 | Bolivia, Brazil, Colombia, Ecuador, Peru |
| *Galbula cyanescens* | Galbulidae | LC | Sta | 1849 | Bolivia, Brazil, Peru |
| *Galbula cyanicollis* | Galbulidae | LC | Decr | 1851 | Bolivia, Brazil, Peru |
| *Galbula pastazae* | Galbulidae | VU | Decr | 1885 | Colombia, Ecuador, Peru |
| *Gastrotheca excubitor* | Hemiphractidae | VU | Decr | 1972 | Peru |
| *Gastrotheca griswoldi* | Hemiphractidae | LC | Sta | 1941 | Peru |
| *Geocerthia serrana* | Furnariidae | LC | Decr | 1875 | Peru |
| *Grallaria andicolus* | Formicariidae | LC | Decr | 1873 | Bolivia, Peru |
| *Grallaria capitalis* | Formicariidae | LC | Sta | 1926 | Peru |
| *Grallaria dignissima* | Formicariidae | LC | Sta | 1880 | Colombia, Ecuador, Peru |
| *Grallaria przewalskii* | Formicariidae | VU | Sta | 1882 | Peru |
| *Graydidascalus brachyurus* | Psittacidae | LC | Decr | 1820 | Brazil, Colombia, Ecuador, French Guiana, Peru |
| *Gymnopithys salvini* | Thamnophilidae | LC | Sta | 1901 | Bolivia, Brazil, Peru |
| *Heliangelus micraster* | Trochilidae | LC | Unkn | 1872 | Ecuador, Peru |
| *Heliangelus strophianus* | Trochilidae | LC | Decr | 1846 | Colombia, Ecuador |
| *Heliangelus viola* | Trochilidae | LC | Sta | 1853 | Ecuador, Peru |
| *Heliodoxa schreibersii* | Trochilidae | LC | Decr | 1847 | Brazil, Colombia, Ecuador, Peru |
| *Hemispingus trifasciatus* | Thraupidae | LC | Decr | 1874 | Bolivia, Peru |
| *Hemispingus xanthophthalmus* | Thraupidae | LC | Decr | 1874 | Bolivia, Peru |
| *Hemitriccus flammulatus* | Tyrannidae | LC | Sta | 1901 | Bolivia, Brazil, Peru |
| *Hemitriccus minor* | Tyrannidae | LC | Sta | 1907 | Bolivia, Brazil, Venezuela |
| *Hemitriccus zosterops* | Tyrannidae | LC | Sta | 1868 | Brazil, Colombia, Ecuador, French Guiana, Guyana, Peru, Suriname, Venezuela |
| *Hylopezus berlepschi* | Formicariidae | LC | Decr | 1903 | Bolivia, Brazil, Peru |
| *Hylopezus fulviventris* | Formicariidae | LC | Decr | 1858 | Colombia, Ecuador, Peru |
| *Hylophilus hypoxanthus* | Vireonidae | LC | Unkn | 1868 | Bolivia, Brazil, Colombia, Ecuador, Peru, Venezuela |
| *Hylophilus olivaceus* | Vireonidae | NT | Decr | 1844 | Ecuador, Peru |
| *Hypocnemis hypoxantha* | Thamnophilidae | LC | Sta | 1869 | Brazil, Colombia, Ecuador, Peru |
| *Hypocnemoides maculicauda* | Thamnophilidae | LC | Decr | 1868 | Bolivia, Brazil, Peru |
| *Hypodactylus nigrovittatus* | Craugastoridae | LC | Sta | 1945 | Colombia, Ecuador, Peru |
| *Hypsiboas fasciatus* | Hylidae | LC | Sta | 1858 | Bolivia, Brazil, Colombia, Ecuador, French Guiana, Guyana, Peru, Suriname |
| *Inezia subflava* | Tyrannidae | LC | Sta | 1873 | Bolivia, Brazil, Colombia, Venezuela |
| *Iridosornis analis* | Thraupidae | LC | Decr | 1844 | Colombia, Ecuador, Peru |
| *Iridosornis jelskii* | Thraupidae | LC | Sta | 1873 | Bolivia, Peru |
| *Iridosornis reinhardti* | Thraupidae | LC | Sta | 1865 | Ecuador, Peru |
| *Isothrix bistriata* | Echimyidae | LC | Unkn | 1845 | Bolivia, Brazil, Colombia, Ecuador, Peru |
| *Lamprospiza melanoleuca* | Thraupidae | LC | Decr | 1817 | Bolivia, Brazil, French Guiana, Guyana, Peru, Suriname |
| *Lanio versicolor* | Thraupidae | LC | Decr | 1837 | Bolivia, Brazil, Peru |
| *Lepidothrix coeruleocapilla* | Pipridae | LC | Sta | 1844 | Peru |
| *Lepidothrix nattereri* | Pipridae | LC | Sta | 1865 | Bolivia, Brazil |
| *Leptasthenura pileata* | Furnariidae | LC | Sta | 1881 | Peru |
| *Leptodactylus pentadactylus* | Leptodactylidae | LC | Sta | 1768 | Bolivia, Brazil, Colombia, Ecuador, French Guiana, Peru |
| *Leptodactylus rhodomystax* | Leptodactylidae | LC | Sta | 1884 | Bolivia, Brazil, Colombia, Ecuador, French Guiana, Guyana, Peru, Suriname |
| *Leptodactylus rhodonotus* | Leptodactylidae | LC | Unkn | 1868 | Bolivia, Brazil, Colombia, Peru |
| *Leptopogon taczanowskii* | Tyrannidae | NT | Decr | 1917 | Peru |
| *Leucippus chlorocercus* | Trochilidae | LC | Unkn | 1866 | Brazil, Colombia, Ecuador, Peru |
| *Leucippus taczanowskii* | Trochilidae | LC | Unkn | 1879 | Peru |
| *Leucopternis kuhli* | Accipitridae | LC | Decr | 1850 | Bolivia, Brazil, Peru |
| *Liosceles thoracicus* | Rhinocryptidae | LC | Sta | 1865 | Bolivia, Brazil, Colombia, Ecuador, Peru |
| *Lophotriccus vitiosus* | Tyrannidae | LC | Sta | 1921 | Brazil, Colombia, Ecuador, French Guiana, Guyana, Peru, Suriname |
| *Malacoptila fulvogularis* | Bucconidae | LC | Sta | 1854 | Bolivia, Colombia, Ecuador, Peru |
| *Marmosops noctivagus* | Didelphidae | LC | Sta | 1844 | Bolivia, Brazil, Ecuador, Peru |
| *Metallura aeneocauda* | Trochilidae | LC | Decr | 1846 | Bolivia, Peru |
| *Metopothrix aurantiaca* | Furnariidae | LC | Decr | 1866 | Bolivia, Brazil, Colombia, Ecuador, Peru |
| *Micrastur buckleyi* | Falconidae | LC | Decr | 1919 | Brazil, Colombia, Ecuador, Peru |
| *Micrastur mintoni* | Falconidae | LC | Decr | 2002 | Bolivia, Brazil |
| *Mitrephanes olivaceus* | Tyrannidae | LC | Sta | 1894 | Bolivia, Peru |
| *Mitu tuberosum* | Cracidae | LC | Decr | 1825 | Bolivia, Brazil, Colombia, Peru |
| *Myioborus melanocephalus* | Parulidae | LC | Decr | 1844 | Bolivia, Colombia, Ecuador, Peru |
| *Myiophobus cryptoxanthus* | Tyrannidae | LC | Increasing | 1860 | Ecuador, Peru |
| *Myiotheretes fuscorufus* | Tyrannidae | LC | Sta | 1876 | Bolivia, Peru |
| *Myrmeciza ferruginea* | Thamnophilidae | LC | Sta | 1776 | Brazil, French Guiana, Guyana, Suriname, Venezuela |
| *Myrmeciza fortis* | Thamnophilidae | LC | Decr | 1868 | Bolivia, Brazil, Colombia, Ecuador, Peru |
| *Myrmeciza goeldii* | Thamnophilidae | LC | Sta | 1908 | Bolivia, Brazil, Peru |
| *Myrmeciza hemimelaena* | Thamnophilidae | LC | Sta | 1857 | Bolivia, Brazil, Peru |
| *Myrmeciza hyperythra* | Thamnophilidae | LC | Sta | 1855 | Bolivia, Brazil, Colombia, Ecuador, Peru |
| *Myrmeciza melanoceps* | Thamnophilidae | LC | Decr | 1825 | Brazil, Colombia, Ecuador, Peru |
| *Myrmochanes hemileucus* | Thamnophilidae | LC | Sta | 1866 | Bolivia, Brazil, Colombia, Ecuador, Peru |
| *Myrmotherula iheringi* | Thamnophilidae | LC | Decr | 1914 | Bolivia, Brazil, Peru |
| *Myrmotherula longicauda* | Thamnophilidae | LC | Decr | 1894 | Bolivia, Colombia, Ecuador, Peru |
| *Myrmotherula sclateri* | Thamnophilidae | LC | Decr | 1912 | Bolivia, Brazil, Peru |
| *Nannopsittaca dachilleae* | Psittacidae | NT | Decr | 1991 | Bolivia, Brazil, Peru |
| *Nectomys apicalis* | Cricetidae | LC | Sta | 1861 | Bolivia, Brazil, Ecuador, Peru |
| *Neoctantes niger* | Thamnophilidae | LC | Decr | 1859 | Brazil, Colombia, Ecuador, Peru |
| *Nephelomyias ochraceiventris* | Tyrannidae | LC | Sta | 1873 | Bolivia, Peru |
| *Nonnula brunnea* | Bucconidae | LC | Decr | 1881 | Colombia, Ecuador, Peru |
| *Nonnula ruficapilla* | Bucconidae | LC | Sta | 1844 | Bolivia, Brazil, Peru |
| *Notharchus macrorhynchos* | Bucconidae | LC | Sta | 1788 | Brazil, French Guiana, Guyana, Suriname, Venezuela |
| *Nothocercus nigrocapillus* | Tinamidae | VU | Decr | 1867 | Bolivia, Peru |
| *Nothocrax urumutum* | Cracidae | LC | Decr | 1825 | Brazil, Colombia, Ecuador, Peru, Venezuela |
| *Nothoprocta curvirostris* | Tinamidae | LC | Sta | 1873 | Ecuador, Peru |
| *Nystalus striolatus* | Bucconidae | LC | Decr | 1856 | Bolivia, Brazil, Ecuador, Peru |
| *Ochthoeca pulchella* | Tyrannidae | LC | Sta | 1876 | Bolivia, Peru |
| *Odontophorus balliviani* | Odontophoridae | LC | Decr | 1846 | Bolivia, Peru |
| *Odontophorus speciosus* | Odontophoridae | NT | Decr | 1843 | Bolivia, Ecuador, Peru |
| *Odontophorus stellatus* | Odontophoridae | LC | Decr | 1843 | Bolivia, Brazil, Ecuador, Peru |
| *Odontorchilus cinereus* | Troglodytidae | NT | Decr | 1868 | Bolivia, Brazil |
| *Oreobates quixensis* | Craugastoridae | LC | Sta | 1872 | Bolivia, Brazil, Colombia, Ecuador, Peru |
| *Oreonympha nobilis* | Trochilidae | LC | Sta | 1869 | Peru |
| *Oreotrochilus chimborazo* | Trochilidae | LC | Sta | 1846 | Colombia, Ecuador |
| *Oreotrochilus melanogaster* | Trochilidae | LC | Sta | 1847 | Peru |
| *Osteocephalus planiceps* | Hylidae | LC | Unkn | 1874 | Colombia, Ecuador, Peru |
| *Osteocephalus verruciger* | Hylidae | LC | Sta | 1901 | Colombia, Ecuador |
| *Parkerthraustes humeralis* | Cardinalidae | LC | Decr | 1867 | Bolivia, Brazil, Colombia, Ecuador, Peru |
| *Patagioenas oenops* | Columbidae | VU | Decr | 1895 | Ecuador, Peru |
| *Percnostola lophotes* | Thamnophilidae | NT | Decr | 1914 | Bolivia, Brazil, Peru |
| *Percnostola rufifrons* | Thamnophilidae | LC | Sta | 1789 | Brazil, Colombia, French Guiana, Guyana, Peru, Suriname, Venezuela |
| *Phaethornis philippii* | Trochilidae | LC | Unkn | 1847 | Bolivia, Brazil, Peru |
| *Phaethornis stuarti* | Trochilidae | LC | Decr | 1897 | Bolivia, Peru |
| *Phalcoboenus carunculatus* | Falconidae | LC | Sta | 1853 | Colombia, Ecuador |
| *Philander andersoni* | Didelphidae | LC | Sta | 1913 | Bolivia, Brazil, Colombia, Ecuador, Peru, Venezuela |
| *Philydor erythrocercum* | Furnariidae | LC | Sta | 1859 | Bolivia, Brazil, Colombia, Ecuador, French Guiana, Guyana, Peru, Suriname |
| *Phlegopsis erythroptera* | Thamnophilidae | LC | Sta | 1855 | Bolivia, Brazil, Colombia, Ecuador, Peru, Venezuela |
| *Phlegopsis nigromaculata* | Thamnophilidae | LC | Decr | 1837 | Bolivia, Brazil, Colombia, Ecuador, Peru |
| *Phoenicircus carnifex* | Cotingidae | LC | Decr | 1758 | Brazil, French Guiana, Guyana, Suriname, Venezuela |
| *Phoenicircus nigricollis* | Cotingidae | LC | Decr | 1832 | Brazil, Colombia, Ecuador, Peru, Venezuela |
| *Phyllomedusa camba* | Hylidae | LC | Sta | 2000 | Bolivia, Brazil, Peru |
| *Phyllomedusa vaillantii* | Hylidae | LC | Sta | 1882 | Bolivia, Brazil, Colombia, Ecuador, French Guiana, Guyana, Peru, Suriname, Venezuela |
| *Phylloscartes orbitalis* | Tyrannidae | LC | Sta | 1873 | Bolivia, Colombia, Ecuador, Peru |
| *Phyllotis andium* | Cricetidae | LC | Sta | 1912 | Ecuador, Peru |
| *Piculus leucolaemus* | Picidae | LC | Decr | 1845 | Bolivia, Brazil, Colombia, Ecuador, Panama, Peru |
| *Picumnus aurifrons* | Picidae | LC | Decr | 1870 | Bolivia, Brazil, Colombia, Peru |
| *Picumnus castelnau* | Picidae | LC | Sta | 1862 | Brazil, Colombia, Ecuador, Peru |
| *Picumnus lafresnayi* | Picidae | LC | Decr | 1862 | Brazil, Colombia, Ecuador, Peru |
| *Pionites leucogaster* | Psittacidae | EN | Decr | 1820 | Brazil |
| *Pipreola chlorolepidota* | Cotingidae | NT | Decr | 1837 | Colombia, Ecuador, Peru |
| *Pipreola frontalis* | Cotingidae | LC | Decr | 1858 | Bolivia, Peru |
| *Pipreola intermedia* | Cotingidae | LC | Sta | 1884 | Bolivia, Peru |
| *Pipreola pulchra* | Cotingidae | LC | Decr | 1917 | Peru |
| *Poecilotriccus albifacies* | Tyrannidae | LC | Decr | 1959 | Peru |
| *Poecilotriccus calopterus* | Tyrannidae | LC | Decr | 1857 | Colombia, Ecuador, Peru |
| *Poecilotriccus capitalis* | Tyrannidae | LC | Decr | 1857 | Brazil, Colombia, Ecuador, Peru |
| *Poospiza alticola* | Emberizidae | EN | Decr | 1895 | Peru |
| *Poospiza caesar* | Emberizidae | LC | Sta | 1869 | Peru |
| *Porphyrolaema porphyrolaema* | Cotingidae | LC | Decr | 1852 | Bolivia, Brazil, Colombia, Ecuador, Peru |
| *Primolius couloni* | Psittacidae | VU | Decr | 1876 | Bolivia, Brazil, Peru |
| *Pristimantis altamazonicus* | Craugastoridae | LC | Sta | 1921 | Brazil, Colombia, Ecuador, Peru |
| *Pristimantis conspicillatus* | Craugastoridae | LC | Sta | 1858 | Brazil, Colombia, Ecuador, Peru |
| *Pristimantis croceoinguinis* | Craugastoridae | LC | Unkn | 1968 | Colombia, Ecuador, Peru |
| *Pristimantis diadematus* | Craugastoridae | LC | Unkn | 1875 | Brazil, Ecuador, Peru |
| *Pristimantis fenestratus* | Craugastoridae | LC | Sta | 1864 | Bolivia, Brazil, Peru |
| *Pristimantis lacrimosus* | Craugastoridae | LC | Unkn | 1875 | Brazil, Colombia, Ecuador, Peru |
| *Pristimantis lanthanites* | Craugastoridae | LC | Sta | 1975 | Brazil, Colombia, Ecuador, Peru |
| *Pristimantis martiae* | Craugastoridae | LC | Sta | 1974 | Brazil, Colombia, Ecuador, Peru |
| *Pristimantis ockendeni* | Craugastoridae | LC | Sta | 1912 | Brazil, Colombia, Ecuador, Peru |
| *Pristimantis peruvianus* | Craugastoridae | LC | Sta | 1941 | Brazil, Colombia, Ecuador, Peru |
| *Pristimantis toftae* | Craugastoridae | LC | Decr | 1978 | Bolivia, Brazil, Peru |
| *Proechimys brevicauda* | Echimyidae | LC | Unkn | 1877 | Bolivia, Brazil, Colombia, Ecuador, Peru, Venezuela |
| *Proechimys simonsi* | Echimyidae | LC | Unkn | 1900 | Bolivia, Brazil, Colombia, Ecuador, Peru |
| *Psarocolius atrovirens* | Icteridae | LC | Sta | 1838 | Bolivia, Peru |
| *Psophia leucoptera* | Psophiidae | NT | Decr | 1825 | Bolivia, Brazil, Peru |
| *Pteroglossus beauharnaesii* | Ramphastidae | LC | Decr | 1832 | Bolivia, Brazil, Peru |
| *Pteroglossus bitorquatus* | Ramphastidae | EN | Decr | 1826 | Brazil |
| *Pulsatrix melanota* | Strigidae | LC | Sta | 1844 | Bolivia, Colombia, Ecuador, Peru |
| *Pyrrhura perlata* | Psittacidae | VU | Sta | 1824 | Bolivia, Brazil |
| *Pyrrhura rupicola* | Psittacidae | NT | Decr | 1844 | Bolivia, Brazil, Peru |
| *Ramphocelus melanogaster* | Thraupidae | LC | Sta | 1838 | Peru |
| *Ramphocelus nigrogularis* | Thraupidae | LC | Sta | 1825 | Bolivia, Brazil, Colombia, Ecuador, Peru |
| *Ramphotrigon fuscicauda* | Tyrannidae | LC | Decr | 1925 | Bolivia, Brazil, Colombia, Ecuador, Peru |
| *Ranitomeya ventrimaculata* | Dendrobatidae | LC | Sta | 1935 | Brazil, Colombia, Ecuador, French Guiana, Peru |
| *Rhegmatorhina melanosticta* | Thamnophilidae | LC | Decr | 1880 | Bolivia, Brazil, Colombia, Ecuador, Peru |
| *Rhinella poeppigii* | Bufonidae | LC | Sta | 1845 | Bolivia, Peru |
| *Saguinus fuscicollis* | Callitrichidae | LC | Decr | 1823 | Bolivia, Brazil, Colombia, Ecuador, Peru |
| *Saguinus imperator* | Callitrichidae | LC | Decr | 1907 | Bolivia, Brazil, Colombia, Peru |
| *Saguinus midas* | Callitrichidae | LC | Sta | 1758 | Brazil, French Guiana, Guyana, Suriname |
| *Saimiri boliviensis* | Cebidae | LC | Decr | 1834 | Bolivia, Brazil, Peru |
| *Saimiri ustus* | Cebidae | NT | Decr | 1843 | Brazil |
| *Sakesphorus luctuosus* | Thamnophilidae | LC | Decr | 1823 | Brazil |
| *Schiffornis major* | Cotingidae | LC | Sta | 1856 | Bolivia, Brazil, Colombia, Ecuador, Peru, Venezuela |
| *Scinax cruentommus* | Hylidae | LC | Sta | 1972 | Brazil, Colombia, Ecuador, Peru |
| *Scinax garbei* | Hylidae | LC | Sta | 1926 | Bolivia, Brazil, Colombia, Ecuador, Peru, Venezuela |
| *Scytalopus acutirostris* | Rhinocryptidae | LC | Sta | 1844 | Peru |
| *Scytalopus femoralis* | Rhinocryptidae | LC | Sta | 1844 | Peru |
| *Scytalopus parvirostris* | Rhinocryptidae | LC | Sta | 1939 | Bolivia, Peru |
| *Selenidera reinwardtii* | Ramphastidae | LC | Unkn | 1827 | Brazil, Colombia, Ecuador, Peru |
| *Sporophila murallae* | Emberizidae | LC | Increasing | 1915 | Brazil, Colombia, Ecuador, Peru |
| *Stefania evansi* | Hemiphractidae | LC | Sta | 1904 | Guyana |
| *Strabomantis sulcatus* | Craugastoridae | LC | Sta | 1874 | Brazil, Colombia, Ecuador, Peru |
| *Synallaxis albigularis* | Furnariidae | LC | Increasing | 1858 | Bolivia, Brazil, Colombia, Ecuador, Peru |
| *Synallaxis cabanisi* | Furnariidae | NT | Decr | 1890 | Bolivia, Brazil, Peru |
| *Synallaxis propinqua* | Furnariidae | LC | Sta | 1859 | Bolivia, Brazil, Colombia, Ecuador, French Guiana, Peru |
| *Tachyphonus rufiventer* | Thraupidae | LC | Sta | 1825 | Bolivia, Brazil, Peru |
| *Tangara callophrys* | Thraupidae | LC | Decr | 1849 | Bolivia, Brazil, Colombia, Ecuador, Peru |
| *Tangara chrysotis* | Thraupidae | LC | Decr | 1846 | Bolivia, Colombia, Ecuador, Peru |
| *Tangara cyanotis* | Thraupidae | LC | Decr | 1858 | Bolivia, Colombia, Ecuador, Peru |
| *Thamnomanes saturninus* | Thamnophilidae | LC | Sta | 1869 | Bolivia, Brazil, Peru |
| *Thamnomanes schistogynus* | Thamnophilidae | LC | Decr | 1911 | Bolivia, Brazil, Peru |
| *Thamnophilus aroyae* | Thamnophilidae | LC | Decr | 1904 | Bolivia, Peru |
| *Thamnophilus cryptoleucus* | Thamnophilidae | NT | Decr | 1906 | Brazil, Colombia, Ecuador, Peru |
| *Thamnophilus schistaceus* | Thamnophilidae | LC | Decr | 1835 | Bolivia, Brazil, Colombia, Ecuador, Peru |
| *Thamnophilus stictocephalus* | Thamnophilidae | LC | Decr | 1869 | Bolivia, Brazil |
| *Thlypopsis inornata* | Thraupidae | LC | Sta | 1879 | Ecuador, Peru |
| *Thlypopsis ornata* | Thraupidae | LC | Sta | 1859 | Colombia, Ecuador, Peru |
| *Thripophaga fusciceps* | Furnariidae | LC | Decr | 1889 | Bolivia, Brazil, Ecuador, Peru |
| *Tinamus guttatus* | Tinamidae | NT | Decr | 1863 | Bolivia, Brazil, Colombia, Ecuador, Peru, Venezuela |
| *Todirostrum chrysocrotaphum* | Tyrannidae | LC | Sta | 1850 | Bolivia, Brazil, Colombia, Ecuador, Peru, Venezuela |
| *Turdus hauxwelli* | Turdidae | LC | Decr | 1869 | Bolivia, Brazil, Colombia, Ecuador, Peru |
| *Turdus maranonicus* | Turdidae | LC | Decr | 1880 | Ecuador, Peru |
| *Veniliornis sanguineus* | Picidae | LC | Sta | 1793 | French Guiana, Guyana, Suriname |
| *Xenopipo unicolor* | Pipridae | LC | Decr | 1884 | Ecuador, Peru |
| *Xiphorhynchus spixii* | Dendrocolaptidae | LC | Sta | 1830 | Bolivia, Brazil, Colombia, Ecuador, Peru |
