# Supplementary material for: Model uncertainties do not affect observed patterns of species richness in the Amazon
Source: PLoS One. 2017 Oct 12;12(10):e0183785. doi: 10.1371/journal.pone.0183785 (PMC5638225; doi:10.1371/journal.pone.0183785)
Supplement: S3 Table — Taxonomic information of species for which we estimated vulnerability to climate change is included. The mean range shift is the variation in the number of suitable climate cells in species potential distribution (current potential distribution—future potential distribution) and was calculated for two scenarios of climate change (rcp26 and rcp85), using IUCN range-maps and point-locality records as input data. (DOCX) [file pone.0183785.s004.docx]

**SUPPORTING INFORMATION**

**Supplementary tables**

**S3 Table.** **Summary statistics of Amazon endemic amphibians, birds and mammals.** Taxonomic information of species for which we estimated vulnerability to climate change is included. The mean range shift is the variation in the number of suitable climate cells in species potential distribution (current potential distribution - future potential distribution) and was calculated for two scenarios of climate change (*rcp*26 and *rcp*85), using IUCN range-maps and point-locality records as input data.

| Species scientific name | Taxonomic group | Mean range shift | | | |
| --- | --- | --- | --- | --- | --- |
|  |  | *rcp*26 | | *rcp*85 | |
|  |  | range-map | point-locality | range-map | point-locality |
| *Aglaeactis castelnaudii* | Bird | -0.88 | -0.71 | -0.99 | -0.82 |
| *Akodon aerosus* | Mammal | -0.79 | -0.63 | -0.90 | -0.69 |
| *Alouatta discolor* | Mammal | -0.73 | -0.24 | -0.88 | -0.44 |
| *Alouatta puruensis* | Mammal | -0.50 | -0.59 | -0.71 | -0.72 |
| *Amazona kawalli* | Bird | -0.69 | -0.74 | -0.84 | -0.84 |
| *Ameerega parvula* | Amphibian | -0.68 | -0.65 | -0.85 | -0.75 |
| *Anabazenops dorsalis* | Bird | -0.68 | -0.64 | -0.84 | -0.72 |
| *Anairetes agraphia* | Bird | -0.55 | -0.69 | -0.77 | -0.80 |
| *Anairetes alpinus* | Bird | -0.65 | -0.71 | -0.85 | -0.89 |
| *Anairetes nigrocristatus* | Bird | -0.63 | -0.42 | -0.82 | -0.65 |
| *Ancistrops strigilatus* | Bird | -0.64 | -0.82 | -0.80 | -0.88 |
| *Anurolimnas castaneiceps* | Bird | -0.51 | -0.72 | -0.73 | -0.82 |
| *Anurolimnas fasciatus* | Bird | -0.87 | -0.63 | -0.98 | -0.73 |
| *Aotus nigriceps* | Mammal | -0.31 | -0.69 | -0.45 | -0.79 |
| *Aotus vociferans* | Mammal | -0.69 | -0.72 | -0.84 | -0.82 |
| *Ara rubrogenys* | Bird | -0.76 | -0.53 | -0.89 | -0.65 |
| *Aratinga weddellii* | Bird | -0.73 | -0.75 | -0.88 | -0.83 |
| *Artibeus anderseni* | Mammal | -0.62 | -0.69 | -0.78 | -0.77 |
| *Asthenes griseomurina* | Bird | -0.58 | -0.46 | -0.76 | -0.51 |
| *Asthenes harterti* | Bird | -0.25 | -0.53 | -0.37 | -0.71 |
| *Asthenes helleri* | Bird | -0.78 | -0.57 | -0.91 | -0.69 |
| *Asthenes humilis* | Bird | -0.44 | -0.68 | -0.57 | -0.75 |
| *Asthenes maculicauda* | Bird | -0.70 | -0.70 | -0.87 | -0.77 |
| *Asthenes ottonis* | Bird | -0.57 | -0.71 | -0.68 | -0.79 |
| *Asthenes urubambensis* | Bird | -0.30 | -0.61 | -0.40 | -0.70 |
| *Asthenes virgata* | Bird | -0.69 | -0.66 | -0.76 | -0.86 |
| *Ateles chamek* | Mammal | -0.42 | -0.57 | -0.59 | -0.80 |
| *Atelopus spumarius* | Amphibian | -0.77 | -0.78 | -0.90 | -0.84 |
| *Atlapetes canigenis* | Bird | -0.52 | -0.84 | -0.63 | -0.90 |
| *Atlapetes melanolaemus* | Bird | -0.26 | -0.64 | -0.36 | -0.77 |
| *Atlapetes rufigenis* | Bird | -0.71 | -0.59 | -0.81 | -0.68 |
| *Atlapetes rufinucha* | Bird | -0.36 | -0.76 | -0.51 | -0.85 |
| *Attila citriniventris* | Bird | -0.65 | -0.74 | -0.82 | -0.81 |
| *Aulacorhynchus coeruleicinctis* | Bird | -0.87 | -0.67 | -0.93 | -0.84 |
| *Automolus melanopezus* | Bird | -0.78 | -0.77 | -0.90 | -0.90 |
| *Boissonneaua matthewsii* | Bird | -0.76 | -0.78 | -0.88 | -0.89 |
| *Brotogeris sanctithomae* | Bird | -0.64 | -0.73 | -0.70 | -0.83 |
| *Callicebus lucifer* | Mammal | -0.61 | -0.71 | -0.77 | -0.84 |
| *Capito aurovirens* | Bird | -0.96 | -0.60 | -0.98 | -0.72 |
| *Cebuella pygmaea* | Mammal | -0.50 | -0.65 | -0.64 | -0.77 |
| *Cercomacra nigrescens* | Bird | -0.15 | -0.46 | -0.31 | -0.66 |
| *Cercomacra serva* | Bird | -0.96 | -0.65 | -0.99 | -0.78 |
| *Certhiaxis mustelinus* | Bird | -0.57 | -0.69 | -0.68 | -0.76 |
| *Chaetura egregia* | Bird | -0.37 | -0.69 | -0.51 | -0.79 |
| *Chalcostigma ruficeps* | Bird | -0.56 | -0.71 | -0.69 | -0.83 |
| *Chalcostigma stanleyi* | Bird | -0.18 | -0.67 | -0.28 | -0.77 |
| *Chamaeza nobilis* | Bird | -0.71 | -0.64 | -0.84 | -0.73 |
| *Chiasmocleis bassleri* | Amphibian | -0.43 | -0.61 | -0.61 | -0.71 |
| *Chiropotes albinasus* | Mammal | -0.63 | -0.55 | -0.79 | -0.73 |
| *Chiropotes satanas* | Mammal | -0.36 | -0.73 | -0.46 | -0.79 |
| *Chlorospingus parvirostris* | Bird | -0.64 | -0.53 | -0.81 | -0.65 |
| *Cinnycerthia fulva* | Bird | -0.03 | -0.32 | -0.18 | -0.62 |
| *Cinnycerthia peruana* | Bird | -0.59 | -0.33 | -0.72 | -0.50 |
| *Cnemarchus erythropygius* | Bird | -0.42 | -0.42 | -0.55 | -0.54 |
| *Coeligena violifer* | Bird | -0.28 | -0.61 | -0.36 | -0.71 |
| *Conioptilon mcilhennyi* | Bird | -0.59 | -0.88 | -0.56 | -0.94 |
| *Conirostrum ferrugineiventre* | Bird | -0.16 | -0.72 | -0.39 | -0.80 |
| *Conopophaga aurita* | Bird | -0.29 | -0.28 | -0.34 | -0.40 |
| *Conopophaga peruviana* | Bird | -0.28 | -0.31 | -0.52 | -0.34 |
| *Cotinga maynana* | Bird | -0.52 | -0.39 | -0.69 | -0.47 |
| *Cranioleuca albicapilla* | Bird | -0.70 | -0.43 | -0.82 | -0.49 |
| *Cranioleuca antisiensis* | Bird | -0.36 | -0.59 | -0.45 | -0.74 |
| *Cranioleuca baroni* | Bird | -0.82 | -0.63 | -0.85 | -0.76 |
| *Cranioleuca marcapatae* | Bird | -0.48 | -0.53 | -0.62 | -0.69 |
| *Cranioleuca vulpecula* | Bird | -0.34 | -0.60 | -0.42 | -0.70 |
| *Creurgops dentatus* | Bird | -0.81 | -0.60 | -0.87 | -0.71 |
| *Crypturellus atrocapillus* | Bird | -0.38 | 0.08 | -0.42 | -0.44 |
| *Crypturellus bartletti* | Bird | -0.76 | -0.18 | -0.89 | -0.32 |
| *Cyanolyca turcosa* | Bird | -0.42 | -0.24 | -0.51 | -0.30 |
| *Cyanolyca viridicyanus* | Bird | -0.65 | -0.61 | -0.81 | -0.72 |
| *Cymbilaimus sanctaemariae* | Bird | -0.55 | -0.31 | -0.68 | -0.37 |
| *Dendroplex kienerii* | Bird | -0.75 | -0.64 | -0.91 | -0.73 |
| *Dendropsophus bifurcus* | Amphibian | -0.57 | -0.50 | -0.74 | -0.62 |
| *Dendropsophus leali* | Amphibian | -0.33 | 0.17 | -0.43 | 0.18 |
| *Dendropsophus leucophyllatus* | Amphibian | -0.40 | -0.43 | -0.53 | -0.60 |
| *Dendropsophus marmoratus* | Amphibian | -0.72 | -0.42 | -0.85 | -0.61 |
| *Dendropsophus rhodopeplus* | Amphibian | -0.43 | -0.48 | -0.64 | -0.60 |
| *Dendropsophus sarayacuensis* | Amphibian | -0.69 | -0.16 | -0.75 | -0.10 |
| *Dichrozona cincta* | Bird | -0.35 | -0.27 | -0.47 | -0.40 |
| *Diglossa glauca* | Bird | -0.37 | -0.81 | -0.43 | -0.91 |
| *Diglossa mystacalis* | Bird | -0.39 | -0.58 | -0.48 | -0.62 |
| *Drymophila devillei* | Bird | -0.91 | -0.66 | -0.99 | -0.77 |
| *Edalorhina perezi* | Amphibian | -0.39 | -0.66 | -0.47 | -0.75 |
| *Elaenia gigas* | Bird | -0.85 | -0.65 | -0.93 | -0.79 |
| *Engystomops petersi* | Amphibian | -0.59 | -0.14 | -0.73 | -0.31 |
| *Entomodestes leucotis* | Bird | -0.70 | -0.22 | -0.83 | -0.34 |
| *Epinecrophylla erythrura* | Bird | -0.39 | -0.26 | -0.82 | -0.38 |
| *Epinecrophylla leucophthalma* | Bird | -0.31 | -0.61 | -0.29 | -0.69 |
| *Epinecrophylla ornata* | Bird | -0.35 | -0.51 | -0.38 | -0.64 |
| *Eriocnemis luciani* | Bird | -0.12 | -0.33 | -0.51 | -0.45 |
| *Eubucco versicolor* | Bird | -0.58 | -0.29 | -0.75 | -0.39 |
| *Euphonia finschi* | Bird | -0.68 | -0.46 | -0.80 | -0.59 |
| *Euphonia mesochrysa* | Bird | -0.87 | -0.27 | -0.96 | -0.29 |
| *Eutoxeres condamini* | Bird | -0.65 | -0.20 | -0.81 | -0.24 |
| *Furnarius minor* | Bird | -0.39 | -0.52 | -0.52 | -0.63 |
| *Galbalcyrhynchus leucotis* | Bird | -0.52 | -0.44 | -0.59 | -0.62 |
| *Galbalcyrhynchus purusianus* | Bird | -0.77 | -0.59 | -0.87 | -0.60 |
| *Galbula chalcothorax* | Bird | -0.79 | -0.68 | -0.93 | -0.70 |
| *Galbula cyanescens* | Bird | -0.71 | -0.13 | -0.83 | -0.12 |
| *Galbula cyanicollis* | Bird | -0.58 | -0.58 | -0.66 | -0.69 |
| *Galbula pastazae* | Bird | -0.51 | -0.26 | -0.62 | -0.36 |
| *Gastrotheca excubitor* | Amphibian | -0.75 | -0.41 | -0.83 | -0.46 |
| *Gastrotheca griswoldi* | Amphibian | -0.64 | -0.24 | -0.73 | -0.31 |
| *Geocerthia serrana* | Bird | -0.56 | -0.47 | -0.64 | -0.59 |
| *Grallaria andicolus* | Bird | -0.48 | -0.70 | -0.57 | -0.80 |
| *Grallaria capitalis* | Bird | -0.80 | -0.55 | -0.91 | -0.71 |
| *Grallaria dignissima* | Bird | -0.74 | -0.40 | -0.85 | -0.45 |
| *Grallaria przewalskii* | Bird | -0.32 | -0.69 | -0.47 | -0.78 |
| *Graydidascalus brachyurus* | Bird | -0.63 | -0.62 | -0.73 | -0.60 |
| *Gymnopithys salvini* | Bird | -0.80 | -0.69 | -0.92 | -0.81 |
| *Heliangelus micraster* | Bird | -0.61 | -0.49 | -0.68 | -0.58 |
| *Heliangelus strophianus* | Bird | -0.57 | -0.35 | -0.66 | -0.45 |
| *Heliangelus viola* | Bird | -0.49 | -0.68 | -0.65 | -0.77 |
| *Heliodoxa schreibersii* | Bird | -0.51 | -0.48 | -0.64 | -0.56 |
| *Hemispingus trifasciatus* | Bird | -0.82 | -0.39 | -0.88 | -0.47 |
| *Hemispingus xanthophthalmus* | Bird | -0.45 | -0.74 | -0.58 | -0.78 |
| *Hemitriccus flammulatus* | Bird | -0.63 | -0.70 | -0.74 | -0.76 |
| *Hemitriccus minor* | Bird | -0.78 | -0.78 | -0.93 | -0.85 |
| *Hemitriccus zosterops* | Bird | -0.71 | -0.63 | -0.92 | -0.79 |
| *Hylopezus berlepschi* | Bird | -0.27 | -0.52 | -0.38 | -0.63 |
| *Hylopezus fulviventris* | Bird | -0.27 | -0.54 | -0.39 | -0.62 |
| *Hylophilus hypoxanthus* | Bird | -0.21 | -0.41 | -0.27 | -0.64 |
| *Hylophilus olivaceus* | Bird | -0.88 | -0.53 | -0.97 | -0.68 |
| *Hypocnemis hypoxantha* | Bird | -0.65 | -0.63 | -0.89 | -0.66 |
| *Hypocnemoides maculicauda* | Bird | -0.67 | -0.54 | -0.79 | -0.61 |
| *Hypodactylus nigrovittatus* | Amphibian | -0.73 | 0.00 | -0.83 | -0.15 |
| *Hypsiboas fasciatus* | Amphibian | -0.23 | -0.79 | -0.26 | -0.84 |
| *Inezia subflava* | Bird | -0.47 | -0.84 | -0.55 | -0.88 |
| *Iridosornis analis* | Bird | -0.25 | -0.43 | -0.36 | -0.56 |
| *Iridosornis jelskii* | Bird | -0.45 | -0.73 | -0.58 | -0.80 |
| *Iridosornis reinhardti* | Bird | -0.40 | -0.73 | -0.48 | -0.85 |
| *Isothrix bistriata* | Mammal | -0.39 | -0.25 | -0.49 | -0.34 |
| *Lamprospiza melanoleuca* | Bird | -0.66 | -0.34 | -0.83 | -0.42 |
| *Lanio versicolor* | Bird | -0.75 | -0.29 | -0.92 | -0.45 |
| *Lepidothrix coeruleocapilla* | Bird | -0.62 | -0.40 | -0.76 | -0.42 |
| *Lepidothrix nattereri* | Bird | -0.69 | -0.17 | -0.85 | -0.22 |
| *Leptasthenura pileata* | Bird | -0.85 | -0.37 | -0.95 | -0.52 |
| *Leptodactylus pentadactylus* | Amphibian | -0.69 | -0.56 | -0.81 | -0.56 |
| *Leptodactylus rhodomystax* | Amphibian | -0.54 | -0.57 | -0.67 | -0.56 |
| *Leptodactylus rhodonotus* | Amphibian | -0.49 | -0.39 | -0.60 | -0.52 |
| *Leptopogon taczanowskii* | Bird | -0.36 | -0.45 | -0.58 | -0.55 |
| *Leucippus chlorocercus* | Bird | -0.73 | -0.66 | -0.91 | -0.77 |
| *Leucippus taczanowskii* | Bird | -0.26 | -0.17 | -0.45 | -0.29 |
| *Leucopternis kuhli* | Bird | -0.35 | -0.48 | -0.46 | -0.62 |
| *Liosceles thoracicus* | Bird | -0.40 | -0.84 | -0.54 | -0.91 |
| *Lophotriccus vitiosus* | Bird | -0.81 | -0.02 | -0.92 | -0.52 |
| *Malacoptila fulvogularis* | Bird | -0.75 | -0.70 | -0.88 | -0.82 |
| *Marmosops noctivagus* | Mammal | -0.59 | -0.44 | -0.68 | -0.50 |
| *Metallura aeneocauda* | Bird | -0.74 | -0.60 | -0.88 | -0.70 |
| *Metopothrix aurantiaca* | Bird | -0.31 | -0.71 | -0.42 | -0.70 |
| *Micrastur buckleyi* | Bird | -0.44 | -0.87 | -0.53 | -0.93 |
| *Micrastur mintoni* | Bird | -0.41 | -0.61 | -0.54 | -0.67 |
| *Mitrephanes olivaceus* | Bird | -0.29 | -0.61 | -0.37 | -0.71 |
| *Mitu tuberosum* | Bird | -0.80 | -0.52 | -0.91 | -0.67 |
| *Myioborus melanocephalus* | Bird | -0.69 | -0.13 | -0.83 | -0.30 |
| *Myiophobus cryptoxanthus* | Bird | -0.60 | -0.48 | -0.69 | -0.58 |
| *Myiotheretes fuscorufus* | Bird | -0.60 | -0.13 | -0.70 | -0.41 |
| *Myrmeciza ferruginea* | Bird | -0.55 | -0.41 | -0.63 | -0.47 |
| *Myrmeciza fortis* | Bird | -0.64 | -0.63 | -0.75 | -0.79 |
| *Myrmeciza goeldii* | Bird | -0.43 | -0.74 | -0.62 | -0.79 |
| *Myrmeciza hemimelaena* | Bird | -0.83 | -0.42 | -0.95 | -0.42 |
| *Myrmeciza hyperythra* | Bird | -0.52 | -0.85 | -0.60 | -0.91 |
| *Myrmeciza melanoceps* | Bird | -0.66 | -0.72 | -0.82 | -0.83 |
| *Myrmochanes hemileucus* | Bird | -0.40 | -0.66 | -0.49 | -0.79 |
| *Myrmotherula iheringi* | Bird | -0.64 | -0.26 | -0.84 | -0.27 |
| *Myrmotherula longicauda* | Bird | -0.48 | -0.46 | -0.49 | -0.66 |
| *Myrmotherula sclateri* | Bird | -0.83 | -0.59 | -0.94 | -0.70 |
| *Nannopsittaca dachilleae* | Bird | -0.63 | -0.55 | -0.78 | -0.79 |
| *Nectomys apicalis* | Mammal | -0.88 | -0.59 | -0.94 | -0.70 |
| *Neoctantes niger* | Bird | -0.63 | -0.45 | -0.77 | -0.49 |
| *Nephelomyias ochraceiventris* | Bird | -0.66 | -0.38 | -0.84 | -0.49 |
| *Nonnula brunnea* | Bird | -0.42 | -0.42 | -0.53 | -0.62 |
| *Nonnula ruficapilla* | Bird | -0.55 | -0.23 | -0.69 | -0.27 |
| *Notharchus macrorhynchos* | Bird | -0.87 | -0.39 | -0.93 | -0.51 |
| *Nothocercus nigrocapillus* | Bird | -0.79 | -0.49 | -0.88 | -0.60 |
| *Nothocrax urumutum* | Bird | -0.86 | -0.69 | -0.92 | -0.76 |
| *Nothoprocta curvirostris* | Bird | -0.74 | -0.70 | -0.84 | -0.87 |
| *Nystalus striolatus* | Bird | -0.49 | -0.15 | -0.55 | -0.36 |
| *Ochthoeca pulchella* | Bird | -0.44 | -0.41 | -0.54 | -0.53 |
| *Odontophorus balliviani* | Bird | -0.45 | -0.40 | -0.62 | -0.53 |
| *Odontophorus speciosus* | Bird | -0.71 | -0.55 | -0.85 | -0.66 |
| *Odontophorus stellatus* | Bird | -0.90 | -0.70 | -0.97 | -0.79 |
| *Odontorchilus cinereus* | Bird | -0.53 | -0.46 | -0.63 | -0.58 |
| *Oreobates quixensis* | Amphibian | -0.74 | -0.50 | -0.84 | -0.72 |
| *Oreonympha nobilis* | Bird | -0.08 | -0.57 | -0.56 | -0.80 |
| *Oreotrochilus chimborazo* | Bird | -0.64 | -0.50 | -0.80 | -0.69 |
| *Oreotrochilus melanogaster* | Bird | -0.45 | -0.59 | -0.58 | -0.68 |
| *Osteocephalus planiceps* | Amphibian | -0.70 | -0.52 | -0.78 | -0.64 |
| *Osteocephalus verruciger* | Amphibian | -0.67 | -0.86 | -0.79 | -0.93 |
| *Parkerthraustes humeralis* | Bird | -0.68 | -0.69 | -0.77 | -0.84 |
| *Patagioenas oenops* | Bird | -0.81 | -0.72 | -0.93 | -0.83 |
| *Percnostola lophotes* | Bird | -0.31 | -0.33 | -0.49 | -0.37 |
| *Percnostola rufifrons* | Bird | -0.56 | -0.52 | -0.64 | -0.63 |
| *Phaethornis philippii* | Bird | -0.41 | -0.43 | -0.57 | -0.53 |
| *Phaethornis stuarti* | Bird | -0.74 | -0.29 | -0.87 | -0.38 |
| *Phalcoboenus carunculatus* | Bird | -0.09 | 0.33 | -0.27 | 0.30 |
| *Philander andersoni* | Mammal | -0.86 | -0.50 | -0.91 | -0.58 |
| *Philydor erythrocercum* | Bird | -0.81 | -0.62 | -0.90 | -0.75 |
| *Phlegopsis erythroptera* | Bird | -0.84 | -0.66 | -0.93 | -0.75 |
| *Phlegopsis nigromaculata* | Bird | -0.78 | -0.79 | -0.84 | -0.86 |
| *Phoenicircus carnifex* | Bird | -0.41 | -0.65 | -0.54 | -0.78 |
| *Phoenicircus nigricollis* | Bird | -0.67 | -0.39 | -0.80 | -0.57 |
| *Phyllomedusa camba* | Amphibian | -0.82 | -0.57 | -0.87 | -0.70 |
| *Phyllomedusa vaillantii* | Amphibian | -0.70 | -0.19 | -0.86 | -0.38 |
| *Phylloscartes orbitalis* | Bird | -0.83 | -0.70 | -0.95 | -0.79 |
| *Phyllotis andium* | Mammal | -0.72 | -0.58 | -0.83 | -0.68 |
| *Piculus leucolaemus* | Bird | -0.64 | -0.72 | -0.74 | -0.85 |
| *Picumnus aurifrons* | Bird | -0.62 | -0.38 | -0.73 | -0.43 |
| *Picumnus castelnau* | Bird | -0.71 | -0.63 | -0.82 | -0.71 |
| *Picumnus lafresnayi* | Bird | 0.54 | -0.50 | 0.51 | -0.78 |
| *Pionites leucogaster* | Bird | -0.44 | -0.53 | -0.58 | -0.71 |
| *Pipreola chlorolepidota* | Bird | -0.66 | -0.44 | -0.81 | -0.62 |
| *Pipreola frontalis* | Bird | -0.54 | -0.70 | -0.76 | -0.75 |
| *Pipreola intermedia* | Bird | -0.55 | -0.62 | -0.62 | -0.77 |
| *Pipreola pulchra* | Bird | -0.46 | -0.22 | -0.51 | -0.28 |
| *Poecilotriccus albifacies* | Bird | -0.26 | -0.83 | -0.39 | -0.96 |
| *Poecilotriccus calopterus* | Bird | -0.79 | -0.73 | -0.90 | -0.85 |
| *Poecilotriccus capitalis* | Bird | -0.85 | -0.54 | -0.92 | -0.69 |
| *Poospiza alticola* | Bird | -0.46 | -0.73 | -0.61 | -0.68 |
| *Poospiza caesar* | Bird | -0.23 | -0.58 | -0.36 | -0.73 |
| *Porphyrolaema porphyrolaema* | Bird | -0.23 | -0.62 | -0.44 | -0.72 |
| *Primolius couloni* | Bird | -0.72 | 0.38 | -0.86 | 0.00 |
| *Pristimantis altamazonicus* | Amphibian | -0.78 | -0.02 | -0.85 | -0.33 |
| *Pristimantis conspicillatus* | Amphibian | -0.70 | -0.43 | -0.77 | -0.44 |
| *Pristimantis croceoinguinis* | Amphibian | -0.69 | -0.38 | -0.84 | -0.57 |
| *Pristimantis diadematus* | Amphibian | -0.68 | -0.73 | -0.82 | -0.86 |
| *Pristimantis fenestratus* | Amphibian | -0.75 | -0.60 | -0.90 | -0.68 |
| *Pristimantis lacrimosus* | Amphibian | -0.66 | -0.49 | -0.74 | -0.67 |
| *Pristimantis lanthanites* | Amphibian | -0.72 | -0.54 | -0.85 | -0.74 |
| *Pristimantis martiae* | Amphibian | -0.71 | -0.78 | -0.79 | -0.85 |
| *Pristimantis ockendeni* | Amphibian | -0.49 | -0.60 | -0.65 | -0.71 |
| *Pristimantis peruvianus* | Amphibian | -0.70 | -0.50 | -0.83 | -0.60 |
| *Pristimantis toftae* | Amphibian | -0.78 | -0.56 | -0.86 | -0.70 |
| *Proechimys brevicauda* | Mammal | -0.55 | -0.71 | -0.65 | -0.80 |
| *Proechimys simonsi* | Mammal | -0.72 | 0.00 | -0.91 | -0.26 |
| *Psarocolius atrovirens* | Bird | -0.70 | -0.65 | -0.81 | -0.75 |
| *Psophia leucoptera* | Bird | -0.87 | -0.44 | -0.93 | -0.52 |
| *Pteroglossus beauharnaesii* | Bird | -0.47 | -0.59 | -0.60 | -0.72 |
| *Pteroglossus bitorquatus* | Bird | -0.59 | -0.55 | -0.68 | -0.64 |
| *Pulsatrix melanota* | Bird | -0.54 | -0.27 | -0.64 | -0.51 |
| *Pyrrhura perlata* | Bird | -0.38 | -0.71 | -0.49 | -0.81 |
| *Pyrrhura rupicola* | Bird | -0.47 | -0.68 | -0.59 | -0.81 |
| *Ramphocelus melanogaster* | Bird | -0.69 | -0.77 | -0.79 | -0.85 |
| *Ramphocelus nigrogularis* | Bird | -0.56 | -0.87 | -0.76 | -0.92 |
| *Ramphotrigon fuscicauda* | Bird | -0.61 | -0.33 | -0.72 | -0.66 |
| *Ranitomeya ventrimaculata* | Amphibian | -0.50 | -0.61 | -0.69 | -0.75 |
| *Rhegmatorhina melanosticta* | Bird | -0.50 | -0.55 | -0.57 | -0.72 |
| *Rhinella poeppigii* | Amphibian | -0.53 | -0.81 | -0.60 | -0.84 |
| *Saguinus fuscicollis* | Mammal | -0.85 | -0.36 | -0.94 | -0.49 |
| *Saguinus imperator* | Mammal | -0.71 | -0.48 | -0.83 | -0.49 |
| *Saguinus midas* | Mammal | -0.88 | -0.79 | -0.96 | -0.92 |
| *Saimiri boliviensis* | Mammal | -0.26 | -0.74 | -0.45 | -0.85 |
| *Saimiri ustus* | Mammal | -0.64 | -0.51 | -0.79 | -0.62 |
| *Sakesphorus luctuosus* | Bird | -0.89 | -0.23 | -0.96 | -0.42 |
| *Schiffornis major* | Bird | -0.43 | -0.06 | -0.45 | -0.25 |
| *Scinax cruentommus* | Amphibian | -0.24 | -0.59 | -0.34 | -0.71 |
| *Scinax garbei* | Amphibian | -0.62 | -0.48 | -0.78 | -0.67 |
| *Scytalopus acutirostris* | Bird | -0.72 | -0.40 | -0.85 | -0.48 |
| *Scytalopus femoralis* | Bird | -0.70 | -0.32 | -0.83 | -0.40 |
| *Scytalopus parvirostris* | Bird | -0.62 | -0.51 | -0.79 | -0.67 |
| *Selenidera reinwardtii* | Bird | 0.14 | -0.55 | -0.04 | -0.63 |
| *Sporophila murallae* | Bird | -0.72 | -0.21 | -0.74 | -0.31 |
| *Stefania evansi* | Amphibian | -0.55 | -0.60 | -0.52 | -0.70 |
| *Strabomantis sulcatus* | Amphibian | -0.86 | -0.66 | -0.97 | -0.68 |
| *Synallaxis albigularis* | Bird | -0.65 | -0.33 | -0.72 | -0.45 |
| *Synallaxis cabanisi* | Bird | -0.63 | -0.73 | -0.75 | -0.82 |
| *Synallaxis propinqua* | Bird | -0.91 | -0.50 | -0.97 | -0.60 |
| *Tachyphonus rufiventer* | Bird | -0.89 | -0.70 | -0.95 | -0.84 |
| *Tangara callophrys* | Bird | -0.85 | -0.74 | -0.92 | -0.84 |
| *Tangara chrysotis* | Bird | -0.50 | -0.73 | -0.65 | -0.85 |
| *Tangara cyanotis* | Bird | -0.66 | -0.75 | -0.80 | -0.81 |
| *Thamnomanes saturninus* | Bird | -0.67 | -0.71 | -0.82 | -0.79 |
| *Thamnomanes schistogynus* | Bird | -0.55 | -0.56 | -0.80 | -0.72 |
| *Thamnophilus aroyae* | Bird | -0.66 | -0.77 | -0.84 | -0.87 |
| *Thamnophilus cryptoleucus* | Bird | -0.85 | -0.45 | -0.95 | -0.56 |
| *Thamnophilus schistaceus* | Bird | -0.88 | -0.68 | -0.97 | -0.81 |
| *Thamnophilus stictocephalus* | Bird | -0.57 | -0.57 | -0.77 | -0.74 |
| *Thlypopsis inornata* | Bird | -0.68 | -0.30 | -0.72 | -0.36 |
| *Thlypopsis ornata* | Bird | -0.51 | -0.31 | -0.61 | -0.37 |
| *Thripophaga fusciceps* | Bird | -0.41 | -0.70 | -0.54 | -0.78 |
| *Tinamus guttatus* | Bird | -0.28 | -0.38 | -0.33 | -0.35 |
| *Todirostrum chrysocrotaphum* | Bird | -0.36 | -0.10 | -0.62 | -0.06 |
| *Turdus hauxwelli* | Bird | -0.62 | -0.70 | -0.79 | -0.73 |
| *Turdus maranonicus* | Bird | -0.59 | -0.53 | -0.71 | -0.63 |
| *Veniliornis sanguineus* | Bird | -0.95 | -0.73 | -0.99 | -0.86 |
| *Xenopipo unicolor* | Bird | -0.84 | -0.68 | -0.94 | -0.82 |
| *Xiphorhynchus spixii* | Bird | -0.61 | -0.56 | -0.75 | -0.74 |
